# Insights Into the Almond Domestication History

**DOI:** 10.1111/eva.70150

**Published:** 2025-08-31

**Authors:** Stephane Decroocq, Amandine Cornille, Naïma Dlalah, Henri Duval, David Tricon, Benedicte Quilot, Wisam K. Khalid, Aurélie Chague, Iban Eduardo, Ignasi Batlle, Pavlina Drogoudi, Ayzin Küden, Bayram M. Asma, Tatiana Kostritsyna, Véronique Decroocq

**Affiliations:** ^1^ INRAE, Biologie du Fruit et Pathologie, UMR 1332, PrADAm Université de Bordeaux Villenave d'Ornon France; ^2^ INRAE, CNRS, AgroParisTech, GQE ‐ IDEEV Université Paris Saclay Gif‐sur‐Yvette France; ^3^ Division of Science New York University Abu Dhabi Saadiyat Island Abu Dhabi UAE; ^4^ GAFL INRAE Montfavet France; ^5^ Department of Horticulture & Landscape Design, College of Agriculture & Forestry University of Mosul Mosul Iraq; ^6^ Centre de Recerca en Agrigenòmica (CRAG), CSIC‐IRTA‐UAB‐UB, Cerdanyola del Vallès (Bellaterra) Institut de Recerca i Tecnologia Agroalimentàries (IRTA) Barcelona Spain; ^7^ Mas Bové Institut de Recerca i Tecnologia Agroalimentàries (IRTA) Constantí Tarragona Spain; ^8^ Department of Deciduous Fruit Trees, Institute of Plant Breeding and Genetic Resources Hellenic Agricultural Organization (ELGO)—‘Dimitra’ Naoussa Greece; ^9^ Department of Horticulture, Faculty of Agriculture Cukurova University (CU) Adana Turkey; ^10^ Department of Horticulture Malatya Turgut Ozal, Inonu University Malatya Turkey; ^11^ International Higher School of Medicine Bishkek Kyrgyz Republic

**Keywords:** almonds, domestication, fruit trees, gene flow, genetic resources, Mediterranean Basin, *Prunus*, sharka, virus

## Abstract

Understanding crop domestication offers crucial insights into the evolutionary processes that drive population divergence and adaptation. It also informs the identification of genetically diverse wild germplasm, which is essential for breeding and conservation efforts. While domestication has been extensively studied in many Mediterranean fruit trees, the evolutionary history of the almond ( *Prunus dulcis* ) remains comparatively underexplored. To address this, we analyzed 209 wild and cultivated almond accessions sampled across Eurasia and genotyped with 23 microsatellite markers. Using population genetics and coalescent‐based inference, we reconstructed the domestication history of *P. dulcis* and its relationships with wild relatives. Bayesian clustering revealed four genetically distinct clusters of cultivated almonds: Turkish, Caucasian–Central Asian, Southern Spanish, and European/North American. These groups were differentiated from wild almond species—including *Prunus turcomanica* , *Prunus orientalis* , *Prunus fenzliana*, and *Prunus spinosissima* —each forming its gene pool across the Middle East and Central Asia. Approximate Bayesian Computation (ABC) supported a single domestication event in the Middle East, originating from either *P. orientalis* or *P. turcomanica* , with subsequent gene flow from *P. fenzliana* and *P. spinosissima* into the Turkish and Central Asian cultivated gene pools, respectively. We also inferred reciprocal introgression from cultivated almonds back into wild populations. Notably, sharka resistance—caused by plum pox virus (PPV)—was identified in three *P. dulcis* clusters and *P. fenzliana*, suggesting that resistance may have arisen independently or been maintained through crop–wild introgression. Together, our results highlight a complex and protracted domestication history for almond, shaped by contributions from multiple wild relatives and recurrent gene flow. These findings enhance our understanding of perennial crop evolution and underscore the value of wild germplasm in breeding programs aimed at increasing resilience in fruit trees.

## 1 Introduction

The study of crop domestication is crucial to unravel the evolutionary processes that shape population divergence (Gaut et al. 2015; Glémin and Bataillon 2009; Meyer et al. 2012) and to design future redomestication strategies (Gradziel 2020) in a context of rare archaeobotanical remains. Documentation on the domestication of fruit tree crops still lags behind that of annual crop plants (Burgarella et al. 2019; Gaut et al. 2015; Meyer et al. 2012; Miller and Gross 2011; Neale et al. 2017). Differences in life‐history traits have likely led to significant differences in the mode and speed of evolution between trees and annuals (Gaut et al. 2015), particularly in the extent of gene flow during domestication and the absence of a bottleneck. Improving knowledge on the domestication of fruit tree crops is important for almond breeding and conservation of genetic resources. Due to long‐term cultivation, fruit trees are increasingly susceptible to the ever‐changing environments caused by climate change and pest outbreaks. In this context, resilient fruit production is believed to rely heavily on untapped wild genetic diversity (Fangning and Jacqueline 2020; Zhang et al. 2017). Such wild germplasm can occur in the center(s) of domestication of crop species, as for apricot (Groppi et al. 2021), peach (Bao et al. 2017), and apple (Cornille et al. 2012; Duan et al. 2017) trees in Central Asia, and for olive trees in the Mediterranean basin (Besnard et al. 2017; Gros‐Balthazard et al. 2019). Wild genetic diversity can also be found and is endemic in additional areas out of the center of origin (Chen, Avia, et al. 2023; Chen, Cornille, et al. 2023; Cornille et al. 2012; Xiao et al. 2023).

The history of the domestication of the almond trees is far less documented than that of other Mediterranean fruit trees. The olive, grape, and date palm domestication histories have been extensively studied (Besnard et al. 2017; Dong et al. 2023; Flowers et al. 2019; Pérez‐Escobar et al. 2021). The cultivated almond ( *Prunus dulcis* (Mill.) D.A. Webb) is an iconic species in Mediterranean and Middle Eastern history, primarily grown for its nuts, which are commercialized worldwide. Indeed, the first step in almond domestication was the selection of almonds with sweet and large kernels, as opposed to the wild almonds with bitter and small kernels (Alioto et al. 2020; Sánchez‐Pérez et al. 2019). This fruit tree crop is an economic pillar of Mediterranean countries and is considered one of the five founders of the Near East Horticulture era, together with olive, grapevine, fig, and date palm (Zohary et al. 2012). Almonds belong to the genus *Prunus* of the Rosaceae family and the Prunoideae subfamily. Among the *Prunus* species, the subgenus Amygdalus includes two sections: Persica (with the peach and peach‐related species) and Amygdalus (with the almond and almond‐related species). The section Amygdalus is a monophyletic group of up to 26 described almond species that occur as trees or shrubs in Central Asia, the Caucasus, the Middle East, and the Mediterranean basin (Browicz and Zohary 1996; Rahemi and Gradziel 2024). Wild almond species are distributed over a large area of over 6000 km in longitude and between 25° and 45° north latitude (Grasselly 1976), which includes severe environments. Adaptation to harsh environmental conditions in almond species is possible due to their genetic and associated developmental and physiological diversity, which is promoted by their typically self‐sterile yet interspecifically fertile compatibility. In almond and stone fruit‐related species, most sources of resistance to sharka, powdery mildew, green peach aphid, brown rot, and root‐knot nematodes have been identified in wild species (Esmenjaud et al. 2009; Marimon et al. 2020; Oliveira Lino et al. 2016; Pascal et al. 2017; Pascal, Pfeiffer, and Kervella 2002; Pascal, Pfeiffer, Kervella, Lacroze, et al. 2002). The high potential of the wild almond species as sources of resistance enhances the interest in almond germplasm in stone fruit breeding programs.

Identifying the wild species that have contributed to the cultivated almond gene pool remains a topic of intense investigation. Hypotheses range from an origin in the Middle East or Central Asia to a complex scenario involving recurrent domestications and crop‐wild gene flow. Domesticated almonds may have originated from the Middle East's wild forms of *P. dulcis*, which were once abundant in the Levantine countries (Lebanon, Israel, Jordan, Palestine, and Syria). Other authors argue that the forms of *P. dulcis* that grow spontaneously in the Middle East are wild types that have escaped cultivation, and that almond domestication occurred earlier in Central Asia, where the most significant number of wild almond species and the greatest number of hybrids are found (Browicz and Zohary 1996; Ladizinsky 1999). Another theory suggests that almond domestication was diffuse, with multiple domestication events involving recurrent wild‐crop genetic exchanges from several wild species that contributed to the current domesticated almond genetic pool (Delplancke et al. 2013). The domestication scenarios of the almond are, therefore, still controversial. However, some wild almond species distributed in the Middle East, the Caucasus, and Central Asia are candidates (Browicz and Zohary 1996; Grasselly 1976). Populations of *Prunus orientalis* and *Prunus turcomanica* are described in the Southeast Anatolia region of Turkey, located in the Middle East (Ak et al. 2001). Frequent spontaneous genetic contributions of *P. orientalis* in the Middle East and *P. webbii* in Southern Europe to the cultivated *P. dulcis* suggest recurrent wild‐to‐crop introgressions (Delplancke et al. 2012; Gradziel and Martinez‐Gomez 2013). From morphological features, it was hypothesized that the almond originated from *Prunus fenzliana* in the Caucasus (Ladizinsky 1999). Natural populations of *P. fenzliana* were identified along the eastern border of Turkey, Azerbaijan, and Iran (Tricon et al. 2023). Several wild almond species found in the Caucasus, the Middle East, and Central Asia likely contributed to the cultivated almond gene pool, either as initial progenitors or through recent wild‐to‐crop introgression (gene flow). However, a comprehensive sampling of wild species and *P. dulcis* across Eurasia is still necessary to fully understand the origins and processes of almond domestication. Understanding almond domestication and the genetic relationships between wild germplasm and the cultivated pool could also shed light on the origin of certain pests and diseases affecting almonds. For example, for sharka, one of the main viral diseases affecting *Prunus* cultivated species, different sources of resistance have been identified in Amygdalus germplasm (Pascal, Pfeiffer, and Kervella 2002; Tricon et al. 2023). However, it remains unclear whether they are phylogenetically related or if they represent distinct evolutionary origins.

In the current study, we investigated the domestication history of almonds using a comprehensive collection of almond cultivars ( *P. dulcis* ) and their wild almond relatives (i.e., *P. fenzliana*, *P. orientalis* , *P. turcomanica* , *P. spinosissima* ), which were genotyped for 23 microsatellite markers. Using population genetics, we explored (i) the population genetic diversity and structure, and genetic relationship among cultivated and wild almond relatives, (ii) the origin of the cultivated almonds and gene flow between crop and wild populations, and (iii) the variability of origin of resistance to sharka in almonds.

## 2 Materials and Methods

### 2.1 Plant Material

Wild almond samples were collected from adult trees between 2008 and 2012 in Azerbaijan, Kyrgyzstan, and Turkey (Eastern Anatolia along the Euphrates River and on the Akdamar Island of the Van Lake). *Prunus spinosissima* was sampled in 2019, after obtaining all local sampling and export authorizations. Almond cultivars used in this study are maintained at INRAE Bordeaux (UMR BFP), INRAE Avignon (GAFL), IRTA‐Mas Bové research station (Constantí, Spain), ELGO‐DIMITRA (Greece), and CU (Turkey).

In total, 209 samples of cultivated and wild almond species were used in this study (Table S1). For the cultivated almond, that is, *P. dulcis* , the 158 accessions originated from 14 countries as follows: Central Asia (Kyrgyzstan, Turkmenistan, and Uzbekistan, *N* = 8), the Caucasus (Azerbaijan and Turkey, *N* = 43), Russia (*N* = 2), Middle East (Israel, *N* = 1), Europe (*N* = 78), North Africa (Tunisia, *N* = 1), and North America (United States, *N* = 16), plus nine accessions from advanced breeding programs (INRAE). A total of nine wild almond‐related species were also sampled: *P. bucharica* (*N* = 1), *P. communis* (*N* = 5), *P. fenzliana* (*N* = 20), *P. kuramica* (*N* = 1), *P. orientalis* (*N* = 9), *P. pedunculata* (*N* = 1), *P. spinosissima* (*N* = 8), *P. turcomanica* (*N* = 5), and *P. webbii* (*N* = 1) (Table S1). *P. communis* , *P. orientalis* , and *P. turcomanica* are native to Turkey; *P. fenzliana* is from Azerbaijan; *P. kuramica* is from Pakistan; *P. pedunculata* is from Mongolia, and *P. spinosissima* is from Kyrgyzstan. *P. bucharica* and *P. webbii* were provided by the ARS‐USDA repository (Davis, USA), but their origin remains unknown.

### 2.2 DNA Extraction and SSR Genotyping

Genomic DNA was extracted from leaves (Decroocq et al. 2016). Twenty‐three microsatellite markers, distributed across the eight chromosomes of *P. dulcis* , *P. persica* , and *P. salicina* , were used for genotyping the 209 accessions (Table S2). Because some markers did not amplify well, the markers with more than 15% missing data across the collection were removed. SSR profiling was scored on an ABI sequencing platform (INRAE, GAFL Avignon). An M13 tail labeling strategy was used, and the fluorochrome LIZ 500 was used as an internal size marker. Raw data were transferred to Bordeaux, and the alleles were then determined and scored by GENEMAPPER v.3.7 software (Applied Biosystems). The bin sets (SSR profiles in Genemapper) for each marker were checked and adjusted according to the fingerprinting of the controls (R190, R269, R61, R40; see Table S1). Additionally, the electropherograms of each sample and each marker were individually checked.

### 2.3 Genetic Variation and Differentiation

Observed and expected heterozygosity (*H*_o_ and *H*_e_, respectively) and the fixation index (*F*_IS_) were calculated with GenAlEx v6.503 (Peakall and Smouse 2012). The significance of pairwise genetic differentiation estimates (*F*_ST_ and Jost's *D*) was assessed with GenAlEx v6.503. The allelic richness (*A*_r_) and the private allelic richness (*A*_p_) were calculated after adjustment for sample size differences among groups (set to four for the *P. dulcis* analysis and to five for the *P. dulcis* and wild‐related species) through the rarefaction procedure implemented in Allelic Diversity Analyzer v1.0 (ADZE [Szpiech et al. 2008]).

#### 2.3.1 Inferences of Population Genetic Subdivision

From the genotyping data, clonemates were identified using GenoDive v.3.04 (Meirmans and Van Tienderen 2004) with a threshold of five. One individual per pair recognized as a clone or sibling was kept.

STRUCTURE v.2.3.4 (Pritchard et al. 2000) without a priori information was used to infer population structure and admixture among wild and cultivated almonds. STRUCTURE is based on Monte Carlo Markov Chain (MCMC) simulations, and it was used to deduce the proportion of ancestry of genotypes in *K* distinct clusters. Firstly, STRUCTURE was run with only cultivated almond trees ( *P. dulcis* , after removal of clonemates and failed DNA extraction, *N* = 138). Secondly, it was run with the above *P. dulcis* , together with wild relative species ( *P. bucharica* , *P. kuramica*, *P. fenzliana*, *P. orientalis* , *P. spinosissima* , *P. turcomanica* , *P. webbii* , *N* = 51). For each *K*, STRUCTURE runs consisted of 10 replicates of 10,000 burn‐in steps followed by 100,000 MCMC iterations. The admixture model with correlated allele frequencies was selected. The resulting matrices of cluster membership coefficients were permuted with CLUMPP v.1.1.2 (Jakobsson and Rosenberg 2007), and bar plots were obtained with DISTRUCT v.1.1 (Rosenberg 2004). The Δ*K* was determined in the online post‐processing software Structure Harvester (Earl and vonHoldt 2012) to determine the strongest level of genetic structure. However, it is possible that the optimal Δ*K* does not reflect the complete subdivision observed in STRUCTURE bar plots. It is, therefore, necessary to compare the obtained bar plots to choose the *K* value for which all clusters have well‐assigned individuals, representing the finest genetic structure and not only the strongest, statistically supported by the Δ*K* (Puechmaille 2016). The problem occurs when the program is forced to cluster individuals into an inappropriately small number of clusters (Cullingham et al. 2020; Puechmaille 2016; Wang 2017) A more powerful method for identifying the best *K* value is to look at the bar plots and choose the highest *K* value for which no clusters are only represented by admixed individuals, indicating that we have reached the highest *K* value for which new genuine clusters could be delimited (Bina et al. 2022; Chen, Avia, et al. 2023; Chen, Cornille, et al. 2023; Cornille et al. 2014, 2012). The *K* value we therefore considered corresponded to the finest one, which can be higher than the *K* value of the strongest population structure level identified by Δ*K*.

Individuals with a membership coefficient ≥ 90% to a *given* cluster were then assigned to a population (i.e., a group of individuals with a membership coefficient ≥ 90% to a given cluster). Individuals with a membership coefficient < 0.90 were considered as admixed (putative hybrids between at least two genetic clusters).

We further explored genetic variation among populations defined above. A factorial correspondence analysis (FCA) was performed using the GENETIX v.4.05 (Belkhir et al. 2004) and then visualized with the “scatterplot3d” R package (Ligges and Mächler 2002). The software DARwin v.6.0.010 (Perrier and Jacquemoud‐Collet 2006) explored genetic differentiation and relationships among samples using a neighbor‐net based on Nei's standard genetic distance (*D*_*st*_) and 30,000 bootstraps.

### 2.4 Approximate Bayesian Computation to Reconstruct the Domestication History of the Almond

The almond domestication history was reconstructed using the approximate Bayesian computation (ABC) framework in combination with the coalescent‐based inference simulator fastsimcoal2 v2.5.2.21 (Excoffier et al. 2021). We aimed to determine (1) whether gene flow occurred among wild populations and crop and wild populations during almond domestication, and (2) from which wild population(s) the cultivated almonds originated. The scenarios were established according to the results obtained from the STRUCTURE, FCA, neighbor‐net tree, *F*_ST_, and Jost's *D* estimates. The populations were defined as those detected with STRUCTURE analyses. Putative hybrids (here referred to as “admixed”) were removed to trace historical gene flow among populations, while more recent admixture events were detected directly from the STRUCTURE analyses. The model parameters used were the divergence time between *X* and *Y* populations (*T*_*X–Y*_), the effective population size of population *X* (*N*_*E‐X*_), and the migration rate per generation between *X* and *Y* populations (*m*_*X–Y*_). Prior values for divergence time were drawn from the log‐uniform distribution bounded between the distributions used in the approximate Bayesian computations, and are listed in Table S3.

A newly developed ABC method was used for model selection and parameter estimation using a machine learning tool called “Random Forest” (ABC‐RF). This approach enabled us to disentangle complex demographic models (Pudlo et al. 2015) by comparing groups of scenarios with a specific type of evolutionary event to other groups with different types of evolutionary events (instead of considering all scenarios separately) (Estoup et al. 2018), which we will hereafter refer to as “ABC rounds.” Such a grouping approach in scenario choice is more powerful than testing all scenarios individually to disentangle the main evolutionary events that characterize speciation (Estoup et al. 2018).

Three nested sets of ABC analyses were conducted. Within each set, two rounds were performed: a first round to test the modalities of gene flow among populations and a second round to investigate the domestication history of the cultivated almonds (Figure S1). For the first ABC set, the modalities of gene flow were as follows: (1) no gene flow, (2) gene flow between crop and wild populations, and (3) gene flow between crop and wild populations only. For the two other ABC sets, there were (1) no gene flow or (2) gene flow between the crop and wild populations only. This nested ABC approach avoids the comparison of complex models with numerous populations and parameters (Estoup et al. 2018).

We used ABCtoolbox (Wegmann et al. 2010) with fastsimcoal 2.5 (Excoffier and Foll 2011) to simulate the datasets. For all models, the microsatellite datasets were simulated for 23 markers. We assumed a generalized stepwise model of microsatellite evolution (Slatkin 1995). Mutation rates were allowed to vary across loci with locus‐specific mutation rates, where *μ* is the mutation rate per generation, with a log‐uniform prior distribution for *μ* (10^e‐4^, 10^e‐3^) (Table S3). The other model parameters were drawn from the prior distributions (Table S1). We performed 10,000 simulations per scenario. For each simulation, we calculated three summary statistics per population with arlsumstats v3.5 (Excoffier and Lischer 2010): *H*, the mean heterozygosity across loci, *NGW*, the adjusted mean Garza‐Williamson statistic over populations (Garza and Williamson 2001), the pairwise *F*_ST_ (Weir and Cockerham 1984) and genetic distances (*δμ*)^2^ (Goldstein et al. 1995) between pairs of populations.

We used the abcrf v.1.7.0 R statistical package (Pudlo et al. 2015) to conduct the ABC‐RF analysis. This analysis provides a classification vote representing the number of times a scenario is selected as the best among the *n* trees in the constructed random forest. For each ABC step, we selected the scenario or the group of scenarios with the highest number of classification votes as the best scenario or the best group of scenarios among 500 classification trees (Breiman 2001). We computed the posterior probabilities and prior error rates (i.e., the probability of choosing an incorrect group of scenarios when drawing the model index and parameter values from the priors of the best scenario) over 10 replicate analyses (Estoup et al. 2018) for each ABC step. We also visually verified that the simulated models were compatible with the observed dataset by projecting both datasets onto the first two linear discriminant analysis (LDA) axes (Pudlo et al. 2015) and checking that the observed dataset fell within the cloud of the simulated datasets. We then calculated parameter inferences using the final selected model. The median and 90% confidence intervals (CI, 5%–95%) are given for each model parameter estimate. Note that the ABC‐RF approach includes the model‐checking step performed a posteriori in previous ABC methods.

## 3 Results

Before analysis, four individuals (Turc_114, Vairo from the French and Greek collections, and Occhiorosso‐2) were removed as they showed more than 50% missing data. SSR fingerprinting also revealed that the US143 accession was polyploid (and was thus discarded); it belongs to the *P. pedunculata* species from Mongolia, where almond species displaying a chromosomal number exceeding the expected 2*n* = 16 have been described (up to 2*n* = 88) (Ekimova et al. 2012). Thirty‐two pairs of clones of cultivated almonds were identified by Genodive analysis. Only one of the two (or three) individuals with high genetic relatedness was retained for analysis. In total, 186 individuals (89%) were retained for the subsequent analyses, including 138 cultivated and 48 wild almonds.

Out of the initial 35 microsatellite markers tested in this study, two (UDP98‐408 and CPDCT035) were filtered out because of too high a rate of missing data, null alleles, or unclear genotype peak patterns (Table S2). The neutrality test implemented in Arlequin v3.5 detected no markers that significantly deviated from a neutral equilibrium model. In particular, marker CPDCT035 did not amplify in *P. fenzliana* samples. Although a majority of the initial genetic markers (23 out of 25, Table S2) were derived from Amygdalus or Persica species, the incidence of missing data or null alleles did not exhibit a significant correlation with the marker's taxonomic origin. This is evidenced by the robust performance of the two Simple Sequence Repeat (SSR) markers originating from *P. salicina* (plum) species (Table S2). In total, we retained 23 microsatellite markers for subsequent analyses of neutral genetic variation and differentiation (Table S2).

### 3.1 Genetic Differentiation and Relationships Among *P. dulcis* and Its Related Species

**FIGURE 1:**
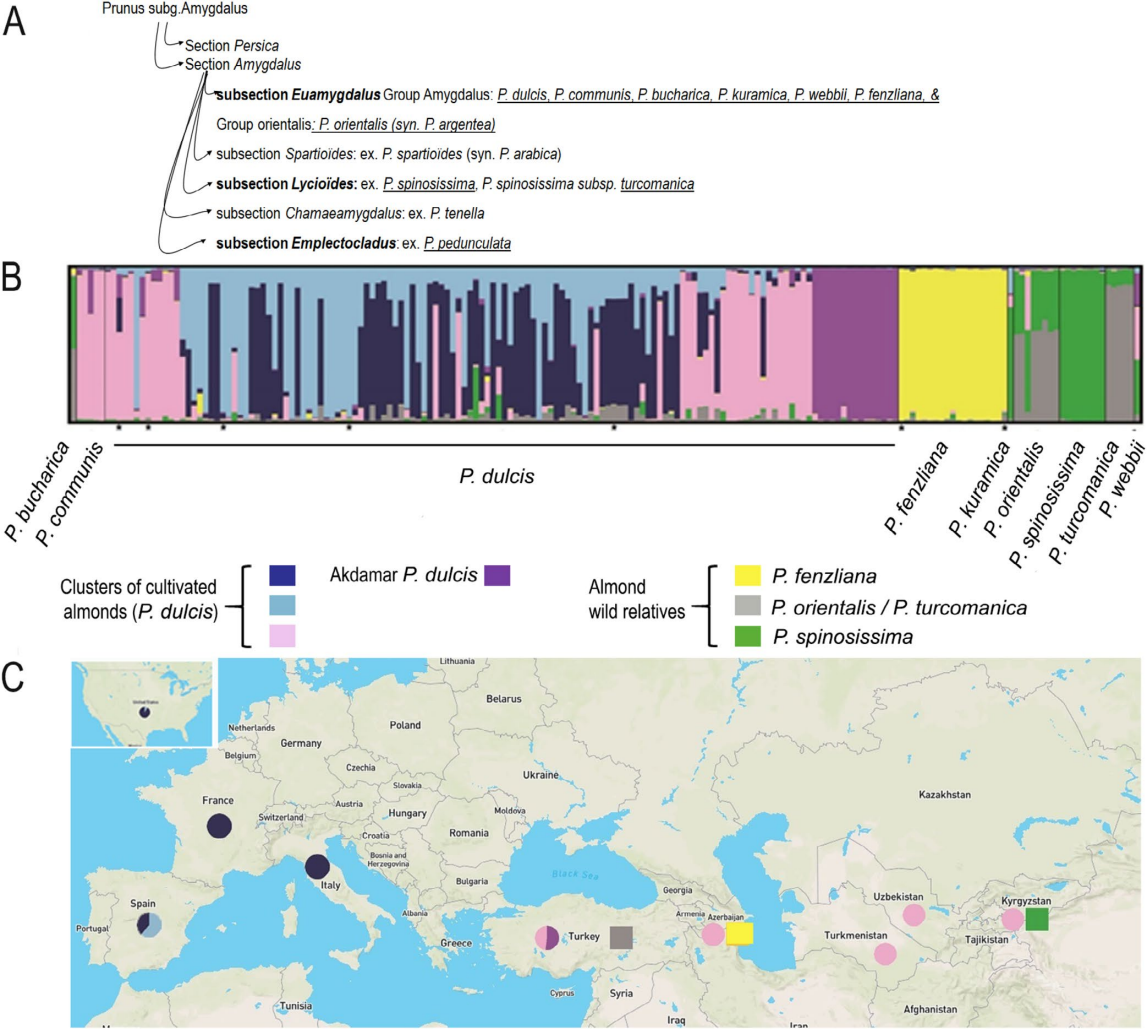
Population genetic structure of *Prunus dulcis* (*N* = 89) and its wild relative species (*Prunus fenzliana*, *N* = 19, *Prunus orientalis*, *N* = 6, *Prunus spinosissima*, *N* = 8, and *Prunus turcomanica*, *N* = 5) inferred with STRUCTURE at *K* = 7. (A) Classification of almond and almond‐related species. Adapted from Browicz and Zohary (1996) and Grasselly (1976). Underlined, species included in this study. (B) Bayesian clustering results inferred with STRUCTURE at *K* = 7. Underneath the figure are depicted the names of the crop and wild Amygdalus populations as described in Table 2. Each individual is represented by a vertical bar, divided into *K* segments representing the amount of ancestry in its genotype corresponding to *K* clusters. (*) refers to Plum Pox virus‐resistant samples as listed in Table S1. Species names are provided primarily as contextual information to relate our genetic clusters to previously identified taxonomic classifications. (C) Spatial population genetic structure of Amygdalus clusters and its wild relatives along the Northern hemisphere. The world map can be downloaded under a free license at https://www.vecteezy.com/vector‐art/10961532‐world‐map‐vector‐illustration‐isolated‐on‐grey‐background‐flat‐earth‐globe‐or‐world‐map.

To characterize the diversity and genetic subdivision among the cultivated almond and related species, we first ran STRUCTURE on the cured dataset (*N* = 186, Table S1bis) composed of cultivated ( *P. dulcis* , *N* = 138) and wild almond‐related species, *P. bucharica* (*N* = 1), *P. communis* (*N* = 5), *P. fenzliana* (*N* = 19), *P. kuramica* (*N* = 1), *P. orientalis* (*N* = 8), *P. spinosissima* (*N* = 8), *P. turcomanica* (*N* = 5), and *P. webbii* (*N* = 1), that were genotyped with 23 microsatellite markers. The change rate in the log‐likelihood between successive *K* values (Δ*K*) inferred with STRUCTURE revealed three peaks at *K* = 2, *K* = 4, and *K* = 7, respectively (Figure S2). At *K* = 4, *P. fenzliana* formed a distinct cluster. The same was true for the *P. dulcis* samples from Akdamar Island (purple color in Figure 1 and Figure S3). A large proportion of the cultivated almonds also formed a distinct cluster (Figure S3). But based on the different bar plots obtained from *K* = 2 to *K* = 10 (Figure S3), we deduced that the most relevant value of *ΔK* is *K* = 7. For *K* < 7, some clusters appeared admixed, while they appeared non‐admixed and well‐delimited at *K* = 7. Further increasing *K* above 7 did not reveal well‐defined new clusters, except for those distinguishing *P. orientalis* and *P. turcomanica* (*K* = 8). However, this differentiation was not stable, as at *K* > 8, the two species were once again indistinguishable (Figure S3). This altogether suggested that *K* = 7 corresponded to the most relevant *K* value for our sampling. The STRUCTURE analysis revealed ambiguous assignments for specific genotypes, particularly those represented by only one or two individuals (e.g., *P. bucharica* , *P. webbii* ). In other cases, such as the grouping of *P. orientalis* and *P. turcomanica* , the observed patterns may reflect either taxonomic misidentification or resolution limitations due to the use of only 23 SSR loci. Additionally, insufficient within‐population sampling can hinder the reliable detection of genetic clusters or gradients (see Puechmaille 2016). For these reasons, we excluded such genotypes from subsequent analyses to ensure more robust and interpretable results.

**FIGURE 2:**
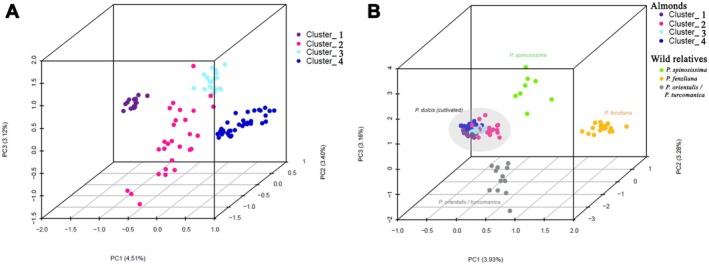
Factorial correspondence analyses (FCAs). (A) Including *Prunus dulcis* individuals (*N* = 98), colored as in Figure 1. (B) Including *P. dulcis* and related species individuals (*N* = 131) with membership coefficient ≥ 90% to a cluster in the STRUCTURE analysis at *K* = 7, colored as in Figure S5. The cluster numbers correspond to (1–4) *P. dulcis* , (5) *P. fenzliana*, (6) *Prunus orientalis* and *Prunus turcomanica*, and (7) *Prunus spinosissima*. Note that individuals with a probability of assignment to one of the seven clusters < 90% were removed.

At *K* = 7, the cultivated almonds split into four distinct clusters (Figure 2A), similar to the ones described above (Figure S3) (pink, purple, light, and dark blue) and the wild‐related species formed two main, non‐admixed clusters, *P. fenzliana* and *P. spinosissima* (yellow and green, respectively in Figures 1A and 2B). The other six wild‐related species were either non‐distinguishable from cultivated *P. dulcis* ( *P. communis* , thus indicating a feral origin), admixed between a wild species and *P. dulcis* ( *P. webbii* , *P. bucharica* , *P. kuramica*, thus indicating a crop‐to‐wild gene flow), or admixed between *P. spinosissima* and another, still‐unknown cluster ( *P. orientalis* and *P. turcomanica* , in gray Figures 1A and 2B). Bayesian clustering thus showed that individuals were mainly classified according to their species and/or origin (for the *P. dulcis* clusters). We confirmed the four genetic clusters of cultivated almonds (*N* = 138) in a separate Bayesian analysis (Data S1, Table S4, Figure S4), and pairwise Jost's *D* among the four cultivated almond clusters were all significant (Table 1).

**TABLE 1:** Pairwise population matrix of Jost's estimate of differentiation (Jost's *D*) among the *Prunus dulcis* clusters inferred with GenAlEx V.6.503.

Jost's *D*			Cluster_1	Cluster_2	Cluster_3	Cluster_4
						
Cluster_1		Akdamar island	—	0.001	0.001	0.001
Cluster_2		Caucasus/Central Asia	0.480	—	0.001	0.001
Cluster_3		South Europe	0.489	0.257	—	0.001
Cluster_4		EU/North‐America	0.552	0.260	0.267	—

*Note:* Only the individuals assigned to a genetic cluster with a membership proportion greater than or equal to 90%, were retained. Jost's *D* values are displayed below the diagonal and the probability above diagonal. All pairwise Jost's *D* values were significant (*p* < 0.05, number of permutations = 999). Colors correspond to genetic clusters identified in Figure 1B.

Subsequently, to estimate the genetic distance between the different almond and almond‐related populations, admixed individuals were removed from the dataset using a 90% assignment threshold to at least one of the seven genetic clusters. At the 90% threshold, a total of 131 non‐admixed accessions were retained as follows: *P. communis* (*N* = 4), *P. dulcis* (*N* = 89), *P. fenzliana* (*N* = 19), *P. orientalis* (*N* = 6), *P. spinosissima* (*N* = 8), and *P. turcomanica* (*N* = 5). The distribution of the almond and almond‐related clusters inferred by Structure (Figure 2B) confirmed a clear spatial differentiation between the four *P. dulcis* clusters and between *P. dulcis* and its wild‐related species following an East to West axis (Figure 1B).

**FIGURE 3:**
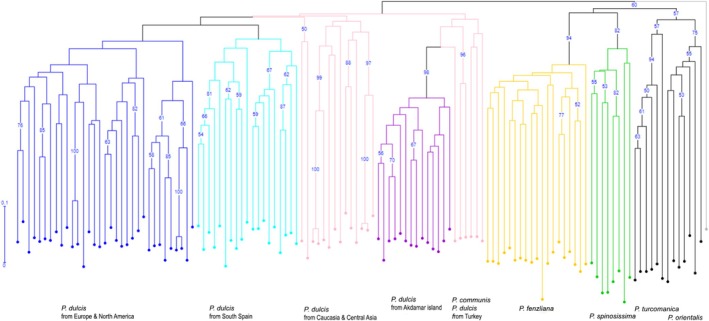
Rooted weighted neighbor‐joining (NJ) tree among cultivated and wild almond species performed with DARwin. The 131 non‐admixed samples belong to six different *Prunus* species including *P. communis* (*N* = 4) in pink and *P. dulcis* (*N* = 89) in pink, light blue, and dark blue, *P. fenzliana* (*N* = 19) in yellow, *P. orientalis* (*N* = 6) in gray, *P. spinosissima* (*N* = 8) in green, and *P. turcomanica* (*N* = 5) in dark gray. In light gray (far right), *P. kuramica* which was used to root the tree. The NJ tree was built with DARwin, bootstrap support values were obtained from 30,000 repetitions. Bootstrap values when greater than 50% are shown above the branches. Species names are provided primarily as contextual information to relate our genetic clusters to previously identified taxonomic classifications.

Further analyses of genetic differentiation between populations (excluding admixed individuals) revealed clear separation between *P. dulcis* and its wild‐related species, as well as among the *P. dulcis* populations (after removing non‐ *P. dulcis* samples). In the FCA (Figure 2B) and neighbor‐joining tree (Figure 3), the wild accessions separated from *P. dulcis* . In the NJ tree, the wild relatives, *P. orientalis* and *P. turcomanica*, were monophyletic but had relatively low bootstrap support (Figure S5). The different wild species clustered together, with a distinct clade for *P. fenzliana*. The overall structure of the dendrogram also aligned with the clustering indicated by STRUCTURE, except for the Caucasian/Central Asian *P. dulcis* cluster (shown in pink in the NJ tree, Figure 3), which split into four main clades.

#### 3.1.1 Genetic Diversity in Wild and Cultivated Almond Populations

As we showed that *P. communis* individuals were most probably feral *P. dulcis* , we grouped the two species, *P. communis* and *P. dulcis*, for subsequent analyses. We then computed population genetic diversity (Table 2) for the five remaining species, excluding *P. webbii* , *P. kuramica*, and *P. bucharica* , as they were represented by a low number of individuals (*N* = 1 or 2 for each). The highest fixation index was that of *P. dulcis* (*F*_IS_ = 0.164, ±0.032, Table 2). However, this value was difficult to compare with the values of the other species due to a significantly unbalanced sample size (i.e., *N* = 92 for *P. dulcis* but *N* = 5 for *P. turcomanica* ). Using ADZE software, we determined the allelic richness of the different populations weighted by the sample size. As a result, *P. fenzliana* not only showed significantly higher allelic richness (*A*_r_ = 4.118 ± 0.138, Table 2) than the other four species, but also the lowest value of unbiased expected heterozygosity (uH_e_ = 0.710 ± 0.051, Table 2).

**TABLE 2:** Genetic diversity estimated for the four cultivated almond populations (*Prunus dulcis*) and three wild species (*Prunus spinosissima*, *Prunus fenzliana*, *Prunus orientalis/turcomanica*) inferred at *K* = 7 with STRUCTURE.

Crop or wild	Species	Populations		*N*	*H*_o_	uH_e_	*F*_IS_	*A*_p_	*A*_r_
Crop	*P. dulcis*	Akdamar island, (purple)		15	0.53	0.56	0.045 NS	0.47 (±0.09)	3.36 (±0.26)
		Caucasus/Central Asia (pink)		23	0.72	0.81	0.11	1.16 (±0.18)	6.06 (±0.40)
		South Europe (light blue)		20	0.73	0.74	0.011 NS	0.63 (±0.10)	4.85 (±0.28)
		EU/North‐America (dark blue)		36	0.66	0.75	0.12	0.45 (±0.09)	4.47 (±0.22)
Wild	*P. spinosissima*	Central Asia (green)		8	0.78	0.85	0.08	2.43 (±0.29)	6.18 (±0.34)
	*P. fenzliana*	Caucasus (yellow)		19	0.64	0.71	0.10	1.79 (±0.27)	4.87 (±0.34)
	*P. orientalis/turcomanica*	Middle East (gray)		12	0.70	0.81	0.13	2.15 (±0.25)	5.83 (±0.36)

*Note:* Only individuals assigned to a genetic cluster with a membership proportion greater than or equal to 90% were retained, that is, 131 accessions. The observed heterozygoty (*H*_o_), the unbiased expected heterozygoty (uH_e_), and the fixation index (*F*_IS_) were calculated thanks to GenAlEx v.6.503, and add‐in off Excel. The allelic richness (*A*_r_) and the private allelic richness (*A*_p_) were calculated thanks to ADZE v.1.0. The standard deviations are depicted in brackets. Species names are provided primarily as contextual information to relate our genetic clusters to previously identified taxonomic classifications.

#### 3.1.2 Approximate Bayesian Computation Analyses for Testing Demographic Scenarios

We defined the populations used in the ABC framework from the clusters detected with STRUCTURE at *K* = 7 (Figure 1) for a total of 131 wild and cultivated almond accessions, excluding admixed individuals (i.e., with a membership coefficient < 0.90 to any given cluster, as recent gene flow can easily be seen from visual inspection of the bar plots). We included *P. spinosissima* , *P. orientalis* , *and P. fenzliana*, two Turkish almond populations (purple and pink), and northwestern cultivated almonds (Spanish and French/North American). The other species were not retained further in the analysis because of their too low number of samples (one or two representatives) or because they were too much admixed and could not be assigned to any population. We also simulated an ancestral population. Eight populations were therefore simulated (Figure S1).

To keep the number of scenarios tractable, we fixed the divergence histories of the wild almond populations and that of the North American/French from the Spanish cultivated almonds as previously known and inferred from the neighbor‐joining tree (Figure S1), *F*_ST_ estimates, and PCA. In the first ABC set, we built three scenarios to test whether the purple Turkish cultivated almond population diverged from one of the three wild populations (Figure S1). These three scenarios were simulated under three alternative hypotheses of gene flow: (1) no gene flow, (2) gene flow between wild populations, and (3) gene flow between crop and wild populations. Thus, nine scenarios (three gene flow hypotheses × three divergence scenarios) were simulated (Figure S1). Once the most likely scenario was chosen, it was used as a backbone for the second ABC set, which tested the origin of the pink Turkish cultivated almond population from one of the three wild populations and the purple Turkish cultivated almond population (three divergence histories, Figure S1). These four scenarios were simulated under two alternative hypotheses of gene flow: (1) no gene flow and (2) gene flow between crop and wild populations only. Once the most likely scenario was chosen, it served as a backbone for the third ABC set, which tested the origin of the Northwestern cultivated almond populations (Spanish and North American/French) from one of the three wild almond populations (three scenarios, Figure S1). Once again, these three scenarios were simulated under two alternative hypotheses of gene flow: (1) no gene flow and (2) gene flow between crop and wild populations only.

For each round of the ABC‐RF approach, the projection of the reference table and the observed datasets onto the two LDA axes that explained most of the variance of the summary statistics showed that the observed data fell within the distribution of the simulated summary statistics (Figures S6–S8), forming different clouds for each scenario or group of scenarios. Visual inspection of the LDA plots indicated that we had enough power to discriminate and select scenarios; the results were subsequently validated by the ABC‐RF inferences presented below.

In the first ABC set, which tested the divergence history of the purple Turkish cultivated almond population, all 10 replicate ABC‐RF analyses supported the occurrence of gene flow only among wild and crop populations (Figure S1, Table S5, an average of 453 votes out of the 500 RF trees; posterior probabilities = 94%, prior error rate = 5.8%) and a sister relationship between the purple Turkish cultivated and *P. orientalis* (Figure S1, Table S6, an average of 292 votes out of the 500 RF trees; posterior probabilities = 68%, prior error rate = 29.49%, Table S9). In the second ABC set, all 10 replicate ABC‐RF analyses supported the occurrence of gene flow among the wild and crop populations (Figure S1, Table S7, an average of 270 out of the 500 RF trees; posterior probabilities = 63%, prior error rate = 14.6%) and a sister relationship between the pink Turkish cultivated and *P. orientalis* (Figure S1, Table S8, an average of 307 votes out of the 500 RF trees; posterior probabilities = 66%, prior error rate = 17.66%). In the third ABC set, all 10 replicate ABC‐RF analyses supported the absence of gene flow among wild and European crop populations (Figure S1, Table S9, an average of 258 out of the 500 RF trees; posterior probabilities = 71%, prior error rate = 2%) and a sister relationship between the northwestern cultivated almonds and *P. orientalis* (Figure S1, Table S10, an average of 357 votes out of the 500 RF trees; posterior probabilities = 75%, prior error rate = 39.21%). This nested ABC approach avoids comparing too many complex models with too many parameters and is more powerful than testing all scenarios individually to disentangle the main evolutionary events characterizing demography and divergence (Estoup et al. 2018).

**FIGURE 4:**
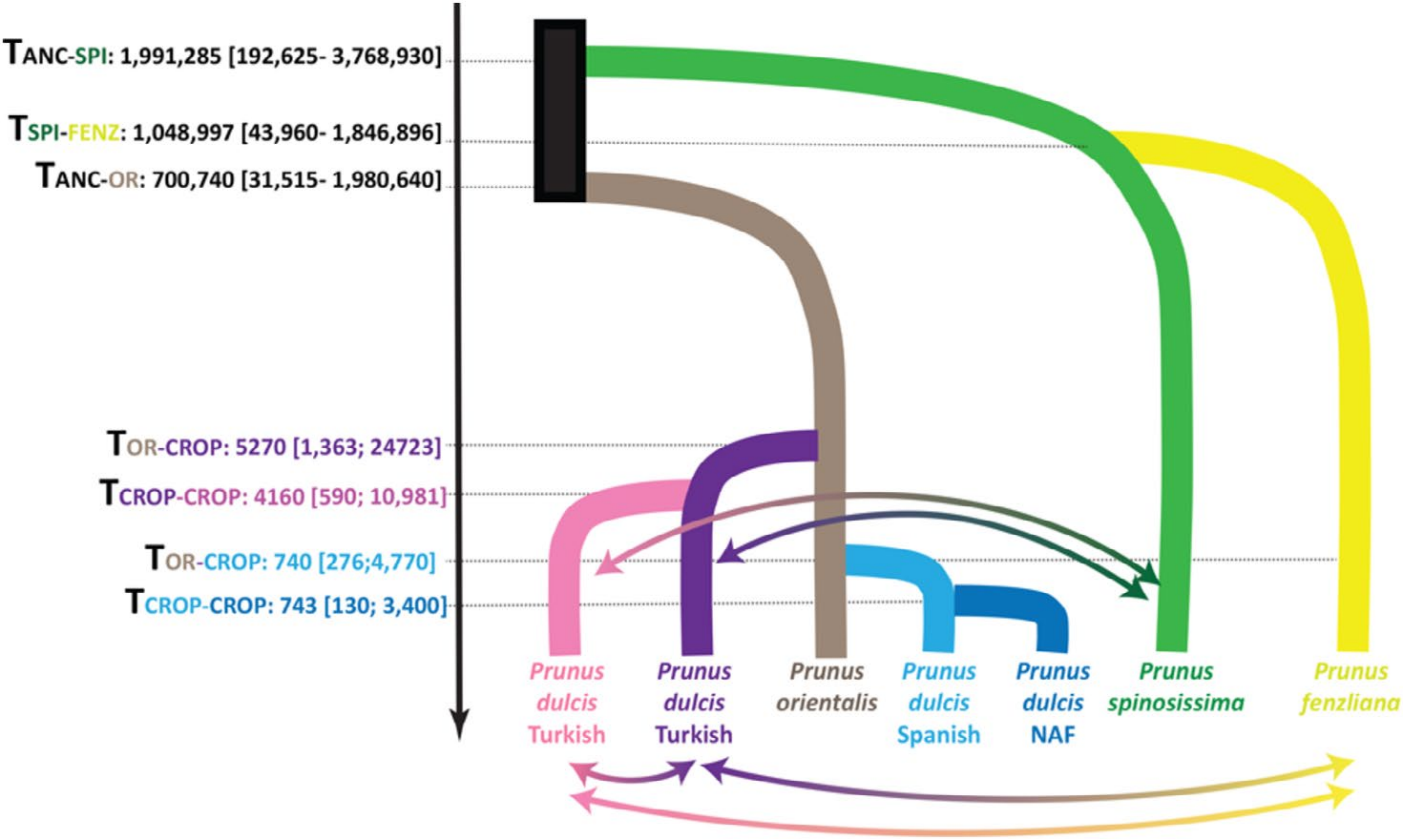
The most likely scenario of domestication of the cultivated *almond* ( *Prunus dulcis* ) with parameter estimates for effective population size and divergence time. The mean posterior estimates for each parameter are written in bold and associated with 95% confidence intervals. The divergence time between populations *X* and *Y*, *T*_*X–Y*_, was provided. *N*_*X*_: effective population size of population *X*; *m*_*X–Y*_: bidirectional arrows represent gene flow between populations *X* and *Y*. Each population detected with STRUCTURE is coded and colored accordingly to Table 2.

Therefore, ABC‐RF inferences provided support for almond domestication events originating from *P. orientalis* only, with substantial gene flow between Turkish cultivated almonds and wild populations, as well as among Turkish cultivated almond populations (Figure 4, Figure S9). However, we found no evidence of gene flow between the northwestern cultivated almond and wild almond populations. The domestication times are presented in Figure 4. Note that the confidence intervals were relatively high for some parameters (e.g., migration rates), as illustrated by the high normalized mean absolute error values (NMAE) (Table S11). Nevertheless, high posterior probabilities and low *prior* error rates for model choice indicate high support for the final model, even if some parameters, such as migration rates, cannot be precisely estimated.

#### 3.1.3 Sharka Resistance in Cultivated and Wild Almonds

In the STRUCTURE barplot (Figure 1B), we indicated (by an asterisk) the Amygdalus accessions that also displayed resistance to plum pox virus after inoculation (Pascal, Pfeiffer, and Kervella 2002; Tricon et al. 2023) (Table S1). The PPV‐resistant almond cultivars tested here were distributed among three different clades of *P. dulcis* (i.e., the Central‐Asian ‐US179‐, Southern European –“Rumbeta” and “Del Cid”‐, and European/North‐American –“Ferragnès,” “Lauranne” and “Mono”‐ almond clusters, corresponding to the pink, light, and dark blue clusters [Pascal, Pfeiffer, and Kervella 2002]). In parallel, several but not all *P. fenzliana* accessions were also found resistant to PPV infection (3 out of 20 tested, i.e., AZ_203_5, AZ_210_6, and AZ_211_10) (Tricon et al. 2023). This would indicate at least four potential sources of resistance to PPV, three in *P. dulcis* and one in *P. fenzliana*.

## 4 Discussion

This study contributes to our understanding of the domestication and diversification of the almond, *P. dulcis* . Our findings suggest a domestication history influenced by contributions from several wild relatives, particularly in the region of present‐day Turkey. This interpretation aligns with archaeobotanical evidence of early *Prunus* cultivation in the Near East (Abbo et al. 2015; Dal Martello et al. 2023), supporting the hypothesis of a complex and gradual domestication process similar to those observed in other perennial fruit crops. However, archaeobotanical records of stone fruits must be taken with caution, as they vary significantly across species, regions, and time periods. Peach and plum remains are the most frequently reported, while many findings are simply classified as *Prunus* sp. This difficulty in identification, along with consumption practices for nuts like almonds, contributes to the unevenness in the archeological record, in particular for *P. dulcis* and related species. ABC analyses indicate gene flow between Turkish almonds and wild populations, while European and North American almonds appear more isolated, likely due to later diffusion and bottleneck effects. Specifically, our ABC modeling supported asymmetric gene flow from wild relatives such as *P. orientalis* and *P. turcomanica* into Turkish *P. dulcis* clusters, with the highest posterior probability corresponding to scenarios involving recurrent introgression after initial domestication. In contrast, gene flow into European and North American clusters was either negligible or not supported across tested models, suggesting long‐term isolation following westward dispersal. The pronounced genetic distinctiveness of the Akdamar population may be attributed to founder effects or localized adaptation. The detection of sharka resistance in several *P. dulcis* clusters and in *P. fenzliana* further underscores the potential value of wild gene pools for breeding. Overall, our results offer a foundation for further investigations into Turkey's role in almond diversification and domestication, and contribute to the growing body of evidence supporting complex domestication histories involving multiple wild taxa and recurrent introgression in perennial crops.

### 4.1 Turkey as a Diversification Center and the Role of Sampling in Resolving Almond Phylogeny

After the divergence between peach and almond that occurred millions of years ago in Central Asia, the almond species dispersed westwards and established itself, particularly in harsh environments, from Central Asia to the Mediterranean area, including the Caucasus and the Middle East (Velasco et al. 2016; Yazbek and Al‐Zein 2014). Previous publications have shown the existence of several almond wild relatives around the Mediterranean Basin; however, none have investigated the relationship between cultivated almonds and their wild relatives due to the lack of a proper sampling design. Indeed, in most cases, authors would compare collections of almond cultivars with only one or two samples per related species (and most often of dubious origin). Lately, we showed in the case of another *Prunus* species, *P. brigantina*, the importance of sample size and sampling design that should encompass sufficient genetic diversity at the species level to result in reliable phylogenetic studies (Liu et al. 2021).

Here, we significantly expanded the sampling of Amygdalus species, which is still limited in some wild species compared to cultivated almonds. It includes worldwide almond cultivars, as well as natural populations of wild relatives from Turkey, the Caucasus, and Central Asia. We identified four genetically differentiated clusters of cultivated almonds ( *P. dulcis* ) with distinct geographical distributions, ranging from Central Asia to Europe and North America. Based on the genotyping of 152 almond landraces with a 60K SNP array, de Pérez los Cobos et al. (2023) confirmed our clustering, but they also identified further subdivisions within the European/North American group (de Pérez los Cobos et al. 2023). In our study, Turkey emerged as a diversification center for cultivated almonds, characterized by two distinct genetic clusters that may have resulted from population isolation and island speciation, as observed in the case of the Akdamar population. This late population would have thus diverged due to local adaptation or reproductive isolation (Westram et al. 2022). Indeed, the case of the Akdamar population must be considered with caution, as its isolation may affect pairwise differentiation and coalescent models. Furthermore, gene flow between the different trees on the island would induce an increase in homozygosity and allele fixation, which could be the cause of the genetic differentiation of this population from the other *P. dulcis* clusters (Halász et al. 2019) reported comparable findings, demonstrating through microsatellite genotyping at 15 loci that Turkish almond accessions resolved into two distinct genetic clusters: one associated with Akdamar Island and another closely related to other Central Asian cultivars. Their study also indicated allele fixation and elevated homozygosity within the Akdamar population. However, contrary to their findings, our investigation did not confirm the presence of Akdamar alleles in the US cultivars.

### 4.2 Geographic Dispersal and Wild–Crop Gene Flow

Besides those two genetic populations in Turkey, we observed two other ones in Europe. One of the two European clusters occurred specifically in Spanish almond cultivars; it may therefore correspond to the Middle Eastern and North African populations (Delplancke et al. 2013). Indeed, after the Muslim conquest of the Iberian Peninsula (711–726 ad), southern Spain has long been a hub of trade between Arabic and European countries (Delplancke et al. 2013). A close relationship between the Southern Spanish and North African gene pools has already been documented for another stone fruit species, apricot (Bourguiba et al. 2012), and more recently for almond (de Pérez los Cobos et al. 2023). It was recently shown that Moroccan almonds present private alleles, most probably resulting from hybridization with *P. arabica* , and thus indicating ancient dissemination routes through Northern Africa toward Spain (Delplancke et al. 2013; Halász et al. 2019). The phylogeographic origin of South Spanish almond cultivars, specifically their potential derivation from a North African gene pool, requires further empirical validation. This necessitates a comparative genetic analysis of both European and North African almond germplasm.

The second European population of cultivated almonds encompasses accessions from North Spain, France, Italy, and Eastern Europe. The identification of a similar sub‐clade of European almonds in prior studies suggests a secondary route for the introduction of *P. dulcis* into Europe, potentially from the East or via ancient Phoenician and Greek trade roads (Delplancke et al. 2013; Halász et al. 2019). For North American individuals, it was not surprising to find them closely related to one of the two European genetic clusters, since all American cultivars originated either directly or after hybridization from more ancient European varieties (de Pérez los Cobos et al. 2021).

We performed Bayesian analysis, highlighting the differentiation between cultivated almond trees and their wild related species. Cluster analysis using SSRs as valuable markers allowed for the discrimination and estimation of relationships among accessions. When wild‐related species are included in the study, *P. dulcis* is still differentiated into four clusters, while *P. communis* samples grouped with the Turkish, Caucasian, and Central Asian *P. dulcis* cultivars and landraces. Moreover, in the phylogenetic tree, *P. communis* individuals were placed in the same clade as *P. dulcis* . We can therefore deduce that the species *P. communis* is a synonym of *P. dulcis* , as previously proposed (Rahemi et al. 2012). Concerning *P. fenzliana*, two independent studies have proposed this species as the wild ancestor of almond (Ladizinsky 1999; Zeinalabedini et al. 2010). According to our phylogenetic analysis, *P. fenzliana* appears as a distinct species, separate from the other Amygdalus species, with limited evidence of admixture. Our phylogenetic study did not support an exclusive relationship between *P. dulcis* and *P. fenzliana*. It showed a kinship between a set of *P. dulcis* cultivars (from Turkey and Caucasus) and the four wild relatives: *P. turcomanica* , *P. orientalis* , *P. spinosissima* , and *P. fenzliana*.

Regarding *P. orientalis* and *P. turcomanica* , it remains unclear at this stage whether they are two distinct species or not. Our Bayesian clustering and phylogenetic results suggest that they are sister species and share a common ancestor. This result is not supported by taxonomic classification, in which *P. orientalis* and *P. turcomanica* belong to two distinct sections: *P. orientalis* to Section *Euamygdalus*, and *P. turcomanica* to *Lycioïdes*. It is also sometimes classified as the *P. spinosissima subspecies turcomanica* (Browicz and Zohary 1996; Grasselly 1976). Indeed, many of the more than 30 named “wild almond” species (Browicz and Zohary 1996; Grasselly 1976; Kester and Gradziel 1996; Kester et al. 1990) may not be true species but interspecific hybrids that then encountered geographic isolation and local adaptation to harsh desert habitats typical of the Central Asian and Middle Eastern center of diversification (Zeinalabedini et al. 2010). Alternatively, the observed intermingling of accessions attributed to *P. orientalis* and *P. turcomanica* in genetic analyses could be attributed to two potential factors: (i) misidentification and/or errors during sample collection or processing, or (ii) insufficient discriminatory power of the employed marker set, specifically the 23 Simple Sequence Repeat (SSR) loci, to resolve the genetic distinctness between these two species adequately.

### 4.3 Sharka Resistance and Its Evolutionary Implications

Although resistance to sharka has been described in the past in almond (Pascal, Pfeiffer, and Kervella 2002; Rubio et al. 2008), its origin and diversity among the Amygdalus species remain poorly understood. To date, no genetic determinant or locus controlling sharka resistance in almond has been published. Thanks to preliminary data on sharka resistance testing, we here questioned the diversity of sources of resistance to sharka in cultivated almonds and their wild relatives. Of the 10 *P. dulcis* accessions identified as resistant to PPV (Pascal, Pfeiffer, and Kervella 2002; Tricon et al. 2023), the PPV‐resistant almond cultivars were distributed among three out of four *P. dulcis* genetic clusters, suggesting multiple origins of the resistance. In addition, we also identified variability in response to sharka among the *P. fenzliana* natural population (Tricon et al. 2023). If the hypothesis of Ladizinsky is true and *P. fenzliana* is the most likely ancestor of *P. dulcis* (Ladizinsky 1999), *P. dulcis* may have inherited the same resistance mechanism from *P. fenzliana*. However, based on Bayesian inference, we showed here a more probable origin of *P. dulcis* from *P. orientalis* than *P. fenzliana*. Nevertheless, without further information on the genetic determinants controlling resistance to PPV in almonds, we cannot rule out the possibility that they share the same resistance mechanism. This preliminary result would benefit from more extensive testing for PPV resistance among the Amygdalus species, as well as from the identification of the genetic factors controlling resistance to sharka in almond. While not indicating the origin of resistance to sharka in almonds, our study is expected to provide valuable sources of resistance, a pool for adaptation to changing environments, and a theoretical basis for understanding the biodiversity and potential of Amygdalus genetic resources.

## Disclosure

Statements: Appropriate permissions from responsible authorities for collecting and using Prunus samples from Central Asia, Caucasia, and Turkey were obtained by the local collaborators, in the frame of the FP7 MSCA IRSES «STONE» project (2011–2014) or the PRIMA FREECLIMB project (2018–2023). Plant material sampled after October 2014, the date of entry in force of the Nagoya protocol, was collected in compliance with the EU Access and Benefit Sharing (ABS) Regulation (EU regulation No 511/2014) and the respective national laws, if any. Due diligence authorizations were submitted online on the DECLARE web‐based application. The rest of the samples were kindly provided by the curators of the French INRAE Genetic Resources Centre (GRC Bourran & Avignon) and the US ARS‐USDA repository; further details are available on their respective databases.

## Ethics Statement

Authors confirm that the manuscript has not been submitted elsewhere and that all research meets the ethical guidelines of their respective countries.

## Conflicts of Interest

The authors declare no conflicts of interest.

## Supporting information


**Data S1:** eva70150‐sup‐0001‐Figures.docx.

**Table S1:** eva70150‐sup‐0002‐Tables.xlsx.

## Data Availability

The datasets generated during the current study, that is, the SSR genotyping, are available at the INRAE data portal (https://entrepot.recherche.data.gouv.fr/dataverse/inrae) where they can be freely retrieved under the link https://doi.org/10.57745/AGTLKC.
