# Supplementary material for: Insights Into the Almond Domestication History
Source: Evol Appl. 2025 Aug 31;18(9):e70150. doi: 10.1111/eva.70150 (PMC12399410; doi:10.1111/eva.70150)
Supplement: Supplementary file 1 — Data S1: eva70150‐sup‐0001‐Figures.docx. [file EVA-18-e70150-s002.docx]

**Supplemental information for: Insights into the almond domestication history**

Stephane Decroocq, Amandine Cornille, Naïma Dlalah, Henri Duval, David Tricon, Benedicte Quilot, Wisam K. Khalid, Aurélie Chague, Iban Eduardo, Ignasi Batlle, Pavlina Drogoudi, Ayzin Küden, Bayram M. Asma, Tatiana Kostritsyna, Véronique Decroocq

# **Supplemental note: *Cultivated almonds (P. dulcis) are subdivided in four main genetic groups***

The second STRUCTURE analysis (cultivated accessions *P. dulcis* only, *N*=138) revealed a clear East-to-West spatial genetic structure. The highest *ΔK* of sample clustering was reached at *K*=3 (Figure S4). However, at *K*=4, *P. dulcis* split into four well-defined genetic clusters among which we retrieved the almond subdivision described by Perez de los Cobos et al (2023), and we, therefore, considered *K*=4 the most likely subdivision for the cultivated almond. At *K*=4, the pink cluster included most individuals from Central Asia and the Caucasus (Figure S4), and the purple included most far Eastern part of Eastern Anatolia cultivated almonds from the Akdamar Island in Turkey (Figure S4). Two other clusters included almond cultivars from Europe and North America (light and dark blue, Figure S4), respectively.

Genotypes were assigned to a given cluster if their membership coefficient for that population was ≥ 0.90. Admixed individuals were removed for further analyses. *F*_IS_ was higher in the dark blue cluster (*F*=0.099, +/-0.049, Table S4) which included European and North American cultivars. We also estimated the Jost's *D* index, in replacement to *F_ST_* as a more accurate measure of genetic differentiation, in case of extreme *F_ST_* values, low and high. One of its particularities is that its maximum values are independent of the average observed heterozygosity, a property that the *F_ST_* does not share (Alcala & Rosenberg, 2019). Jost’s *D* values revealed highly significant genetic differentiation among the *P. dulcis* clusters (*p*< 0.001) (Table 1). The highest degrees of differentiation was observed between Akdamar island *P. dulcis* cluster and the European, North American and Central Asian/Caucasian clusters, while the three last clusters were equally differentiated (Jost’s *D* values around 0.250, Table 1).

The Central Asia and Caucasus genetic cluster (pink) showed the highest level of allelic richness (mean ± standard deviation: A_r_ = 3.791 ± 0.181) and heterozygosity level (mean ± standard deviation: H_e_ = 0.743 ± 0.042, Table S4). The genetic cluster from Eastern Anatolia of the Akdamar island individuals displayed the lowest heterozygosity (mean ± standard deviation: H_e_ = 0.527 ± 0.055, Table S4) and allelic richness (mean ± standard deviation: A_r_ = 2.518 ± 0.166, Table 1, respectively). The purple cluster corresponding to the Akdamar population thus appeared geographically isolated and genetically highly differentiated from the other genetic clusters, most likely contributing to its relative high level of homozygosity.


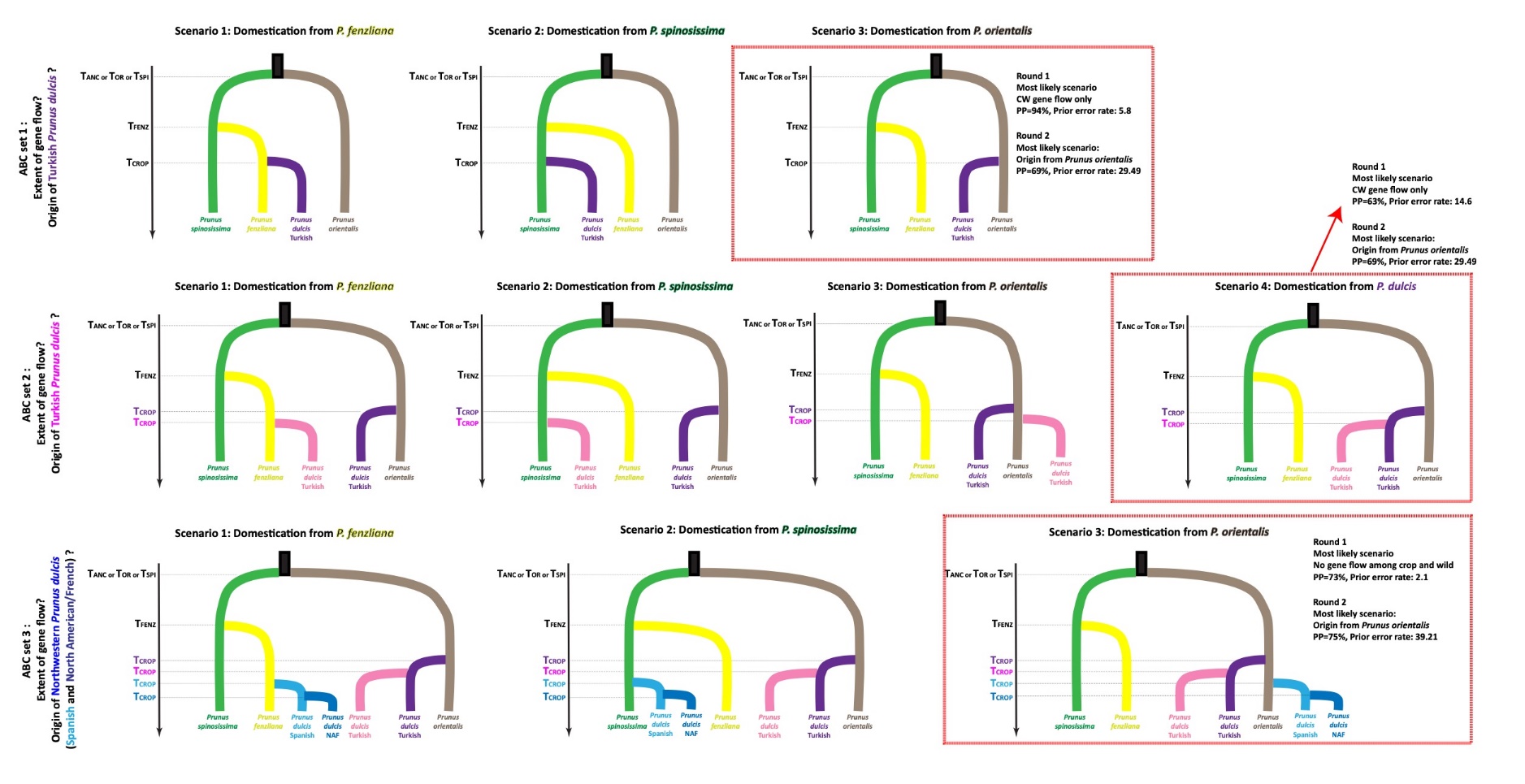


# **Figure S1. Details of the scenarios tested with approximate Bayesian computation for reconstructing almond domestication *(Prunus dulcis).***

The first ABC set inferred the domestication history of the purple Turkish *Prunus dulcis* population, including three scenarios of divergence of the Turkish purple *Prunus dulcis*, simulated with i) no gene flow, ii) bidirectional gene flow between wild and cultivated populations only, and iii) bidirectional gene flow between wild and cultivated populations, and among wild populations*.* The second ABC set inferred the domestication history of the pink Turkish *Prunus dulcis* population, including four scenarios of divergence of the Turkish purple *Prunus dulcis*, simulated with i) no gene flow and ii) bidirectional gene flow between wild and cultivated populations only*.* The third ABC set inferred the domestication history of the blue northwestern *Prunus dulcis* populations (including the Spanish and North American/French populations), including three scenarios of divergence of the blue northwestern *Prunus dulcis* populations, simulated with i) no gene flow and ii) bidirectional gene flow between wild and cultivated populations only. *NAF:* North American/French population*, Prunus dulcis.* The parameters used are listed in Table S3.

**
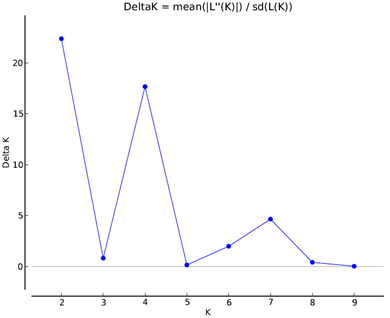
**

# **Figure S2. Δ*K* plotted against *K* values for the *Prunus dulcis* and its wild-related species used to choose the most likely *K* value for STRUCTURE analysis.**

The ΔK was estimated by Structure harvester for nine different Prunus species including P. bucharica (N=1), P. communis (N=5), P. dulcis (N=138), P. fenzliana (N=19), P. kuramica (N=1), P. orientalis (N=8), P. spinosissima (N=8), P. turcomanica (N=5) and P. webbii (N=1). STRUCTURE results are based on 23 microsatellite markers.

*
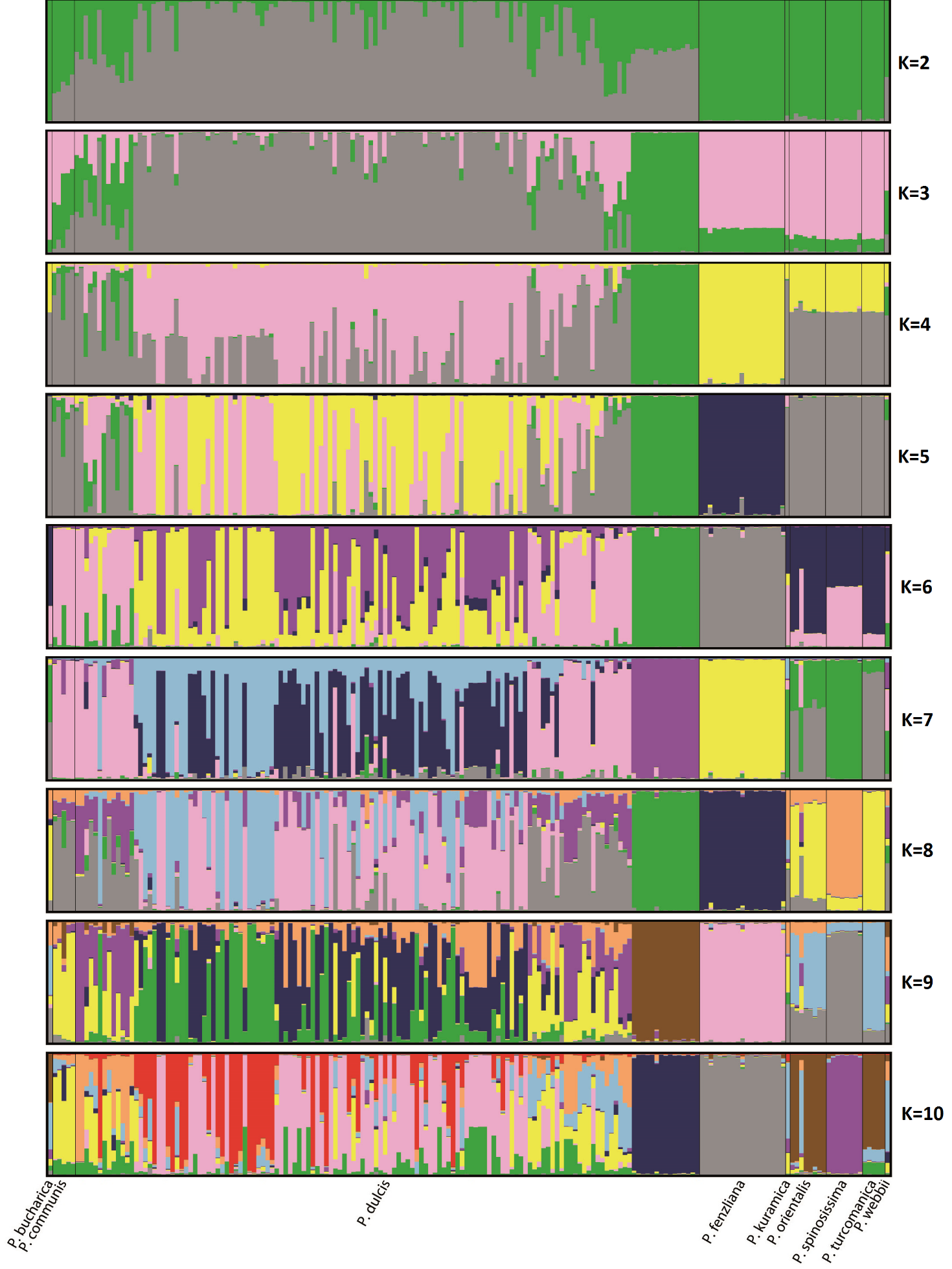
*

# **Figure S3. Genetic subdivision of the cultivated almonds (*Prunus dulcis*) and its wild related species inferred with STRUCTURE from *K*=2 to *K*=10 and with 23 microsatellite markers.**

The 186 individuals belong to nine different *Prunus* species, including *P. bucharica* (*N*=1), *P. communis* (*N*=5), *P. dulcis* (*N*=138), *P. fenzliana* (*N*=19), *P. kuramica* (*N*=1), *P. orientalis* (*N*=8), *P. spinosissima* (*N*=8), *P. turcomanica* (*N*=5) and *P. webbii* (*N*=1). Each individual was represented by a vertical bar, and each color represented the proportion of membership in each genetic group *K* as implemented in STRUCTURE for each individual.

# **
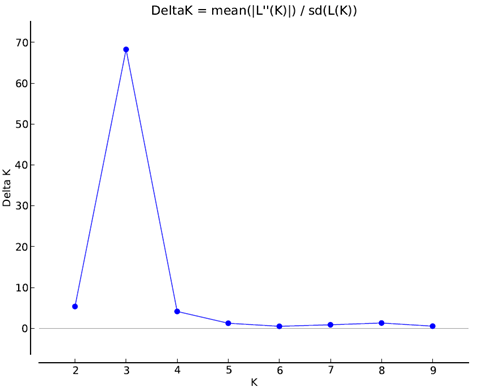

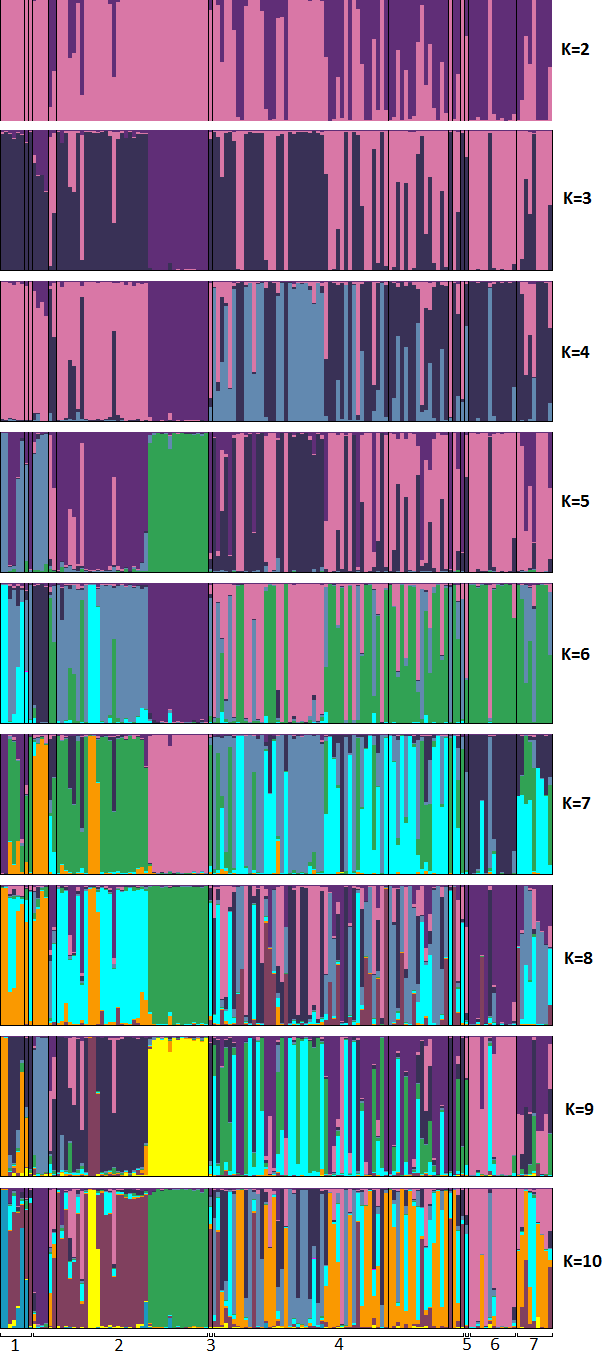
Figure S4: Genetic subdivision of *Prunus dulcis* (*N*=138, 23 microsatellite markers).**

**B**

**A**

**A**- Barplots **inferred with STRUCTURE for *K*=2 to *K*=10** The one hundred and thirty-eight *P. dulcis* accessions include samples from Central Asia (Kyrgyzstan, Turkmenistan and Uzbekistan, *N*=8), Caucasia (Azerbaijan, Russia and Turkey, *N*=44), Middle East (Israel, *N*=1), Europe (Spain, France, Greece, Italy and Portugal, *N*=63), North Africa (Tunisia, *N*=1), North America (United States, *N*=12) and breeding accessions (*N*=9). Each individual was represented by a vertical bar, and each color represented the proportion of membership in each genetic group *K* as implemented in STRUCTURE for each individual. The numbers along the x-axis correspond to (1) Central Asia, (2) Caucasia, (3) Middle East, (4) Europe, (5) North Africa, (6) North America and (7) Modern almond cultivars issued from last century breeding programs.

**B**-The ΔK was estimated by Structure harvester for the P dulcis dataset (N=138) and 23 microsatellite markers.


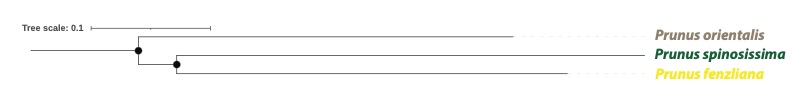


# **Figure S5. Neighbor-net representing the genetic relationships among the three wild almond populations (*Prunus spinosissima*, *Prunus fenzliana*, and *Prunus orientalis*) inferred using STRUCTURE at *K* = 7.**

Colours correspond to the genetic groups inferred with STRUCTURE at *K*=7, and admixed were removed, as well as cultivated almond populations.


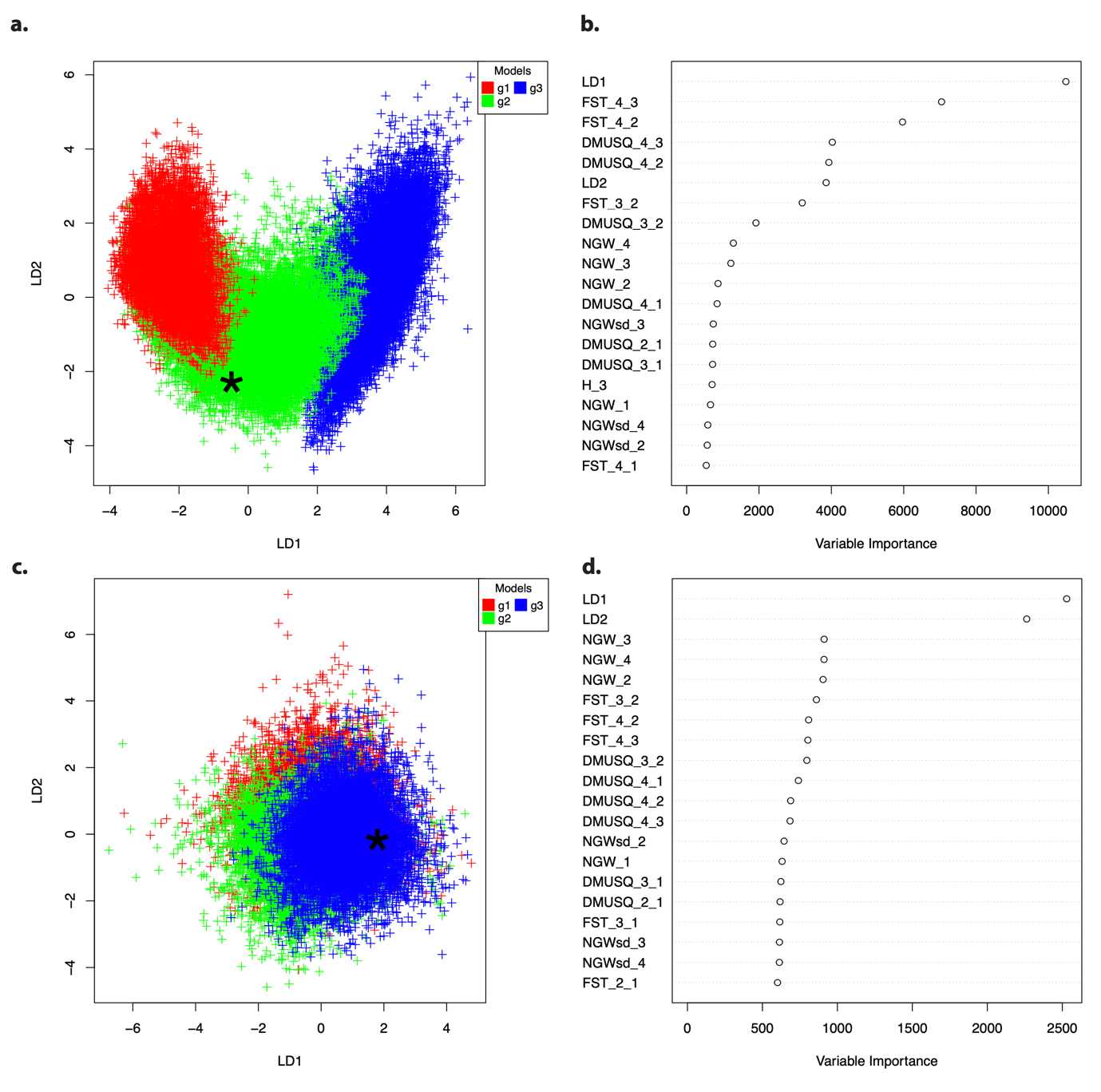


# **Figure S6. Linear discriminant analysis (LDA 1 and 2) and associated variable importance of summary statistics for round 1 “Gene flow test” (a and b), and for round 2 “Origin of the purple Turkish cultivated almond (*Prunus dulcis*)” (c and d).**

The first round included nine scenarios with three scenarios of divergence of the Turkish purple *Prunus dulcis*, simulated with i) no gene flow (group 1), ii) bidirectional gene flow between wild and cultivated populations only (group 2), and iii) bidirectional gene flow between wild and cultivated populations, and among wild populations (group 3). The second round compared the three scenarios of domestication of the purple Turkish *Prunus dulcis*, assuming the most likely scenario of gene flow inferred in round 1 (i.e., gene flow between only crop and wild populations (Table S5 and Figure S1)*.* Each scenario was simulated 10,000 times; the black star represents the observed data and each cross represents one simulation.

**
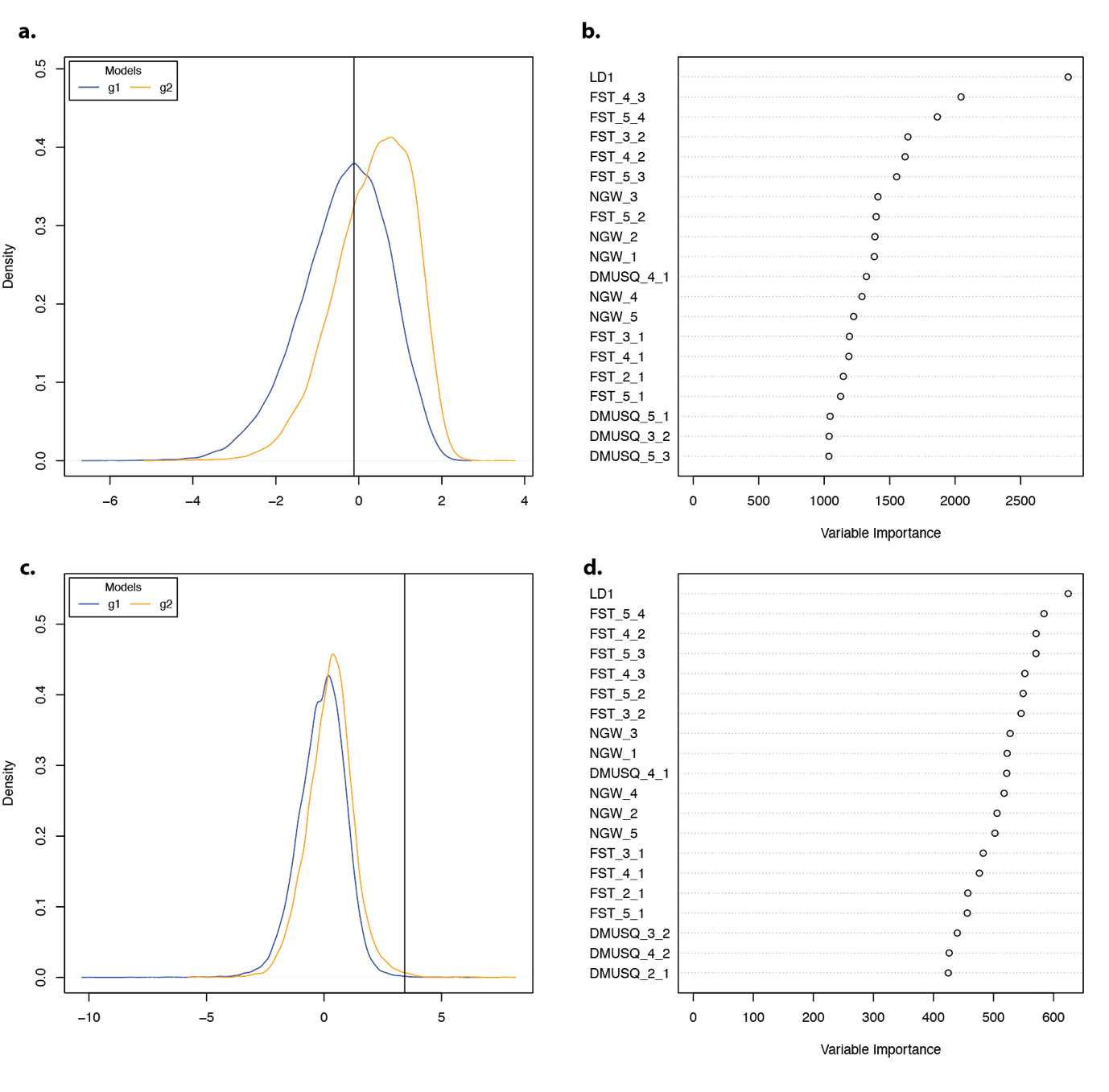
**

# **Figure S7. Linear Discriminant Analyses (LDA 1 and 2) and associated variable importance of summary statistics for round 1 “Gene flow test” (a and b), and for round 2 “Origin of the pink Turkish cultivated almond (*Prunus dulcis*)” (c and d).**

The first round included eight scenarios with four scenarios of divergence of the Turkish pink *Prunus dulcis*, simulated with i) no gene flow (group 1) and ii) bidirectional gene flow among the wild and cultivated populations only (group 2). The second round compared the two groups of domestication scenarios of the Turkish pink *Prunus dulcis:*1) domestication from one of three wild species (*Prunus spinosissima*, *Prunus orientalis*, and *Prunus fenzliana*) or 2) domestication from the purple Turkish *Prunus dulcis*. We assumed the most likely scenario of gene flow inferred in round 1 (i.e., gene flow between only crop and wild populations (Table S7 and Figure S1)*.* Each scenario was simulated 10,000 times; the black line represents the observed data and each cross represents one simulation.

**
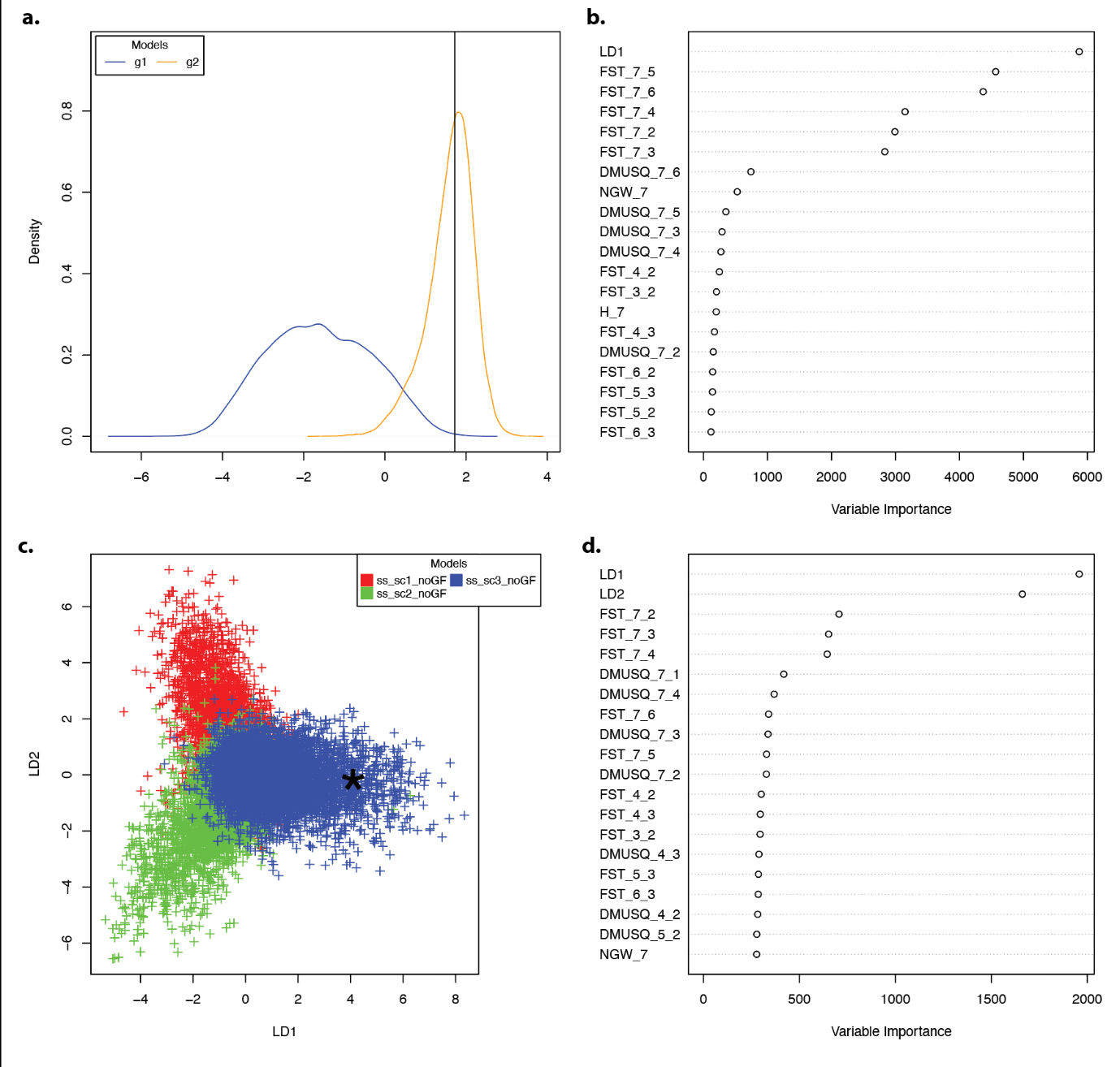
**

**Figure S8.** **Linear Discriminant Analyses (LDA 1 and 2) and associated variable importance of summary statistics for round 1 “Gene flow test” (a and b), and for round 3 “Origin of the blue Northwestern cultivated almonds (*Prunus dulcis*)” (c and d).** The first round included six scenarios with three scenarios of divergence of the blue northwestern cultivated almonds, simulated with i) no gene flow (group 1) and ii) bidirectional gene flow among the wild and cultivated populations only (group 2). The second round compared the three scenarios of domestication of the blue Northwest cultivated almonds *Prunus dulcis:*1) domestication from *Prunus spinosissima*, 2) *Prunus fenzliana*, or 3) *Prunus orientalis*. We assumed the most likely scenario of gene flow inferred in round 1 (i.e., no gene flow between only crop and wild populations (Table S9 and Figure S1)*.* Each scenario was simulated 10,000 times; the black line or star represents the observed data and each cross represents one simulation.

# **Figure S9. Histogram of 1,000 simulations of the most likely scenario of almond domestication history inferred using ABC (*i.e.*, set 3, scenario 3, Figure S1).**

The pseudo-observed dataset was obtained using prior distributions drawn from 90% confidence intervals of the parameters estimated from the most likely model (Tables S8 & S10). This histogram is plotted under H0 (*i.e.*, the simulated dataset fits with the observed dataset), and results obtained with the goodness-of-fit test from the abc R package (Csilléry, François, & Blum, 2012), (*P*=0.05) were barely non-significant, further confirming our model choice.

**Table S2: List of the 25 microsatellite markers used in this study**

Chr: Chromosome where the SSR locus is located in the Prunus reference map (see [www.rosaceae.org](http://www.rosaceae.org) for details)

| **Marker name** | **Sequence-Forward** | **Sequence-Reverse** | **Chr *** | **Origin of the marker** |
| --- | --- | --- | --- | --- |
| UDP96-018 | TTCTAATCTGGGCTATGGCG | GAAGTTCACATTTACGACAGGG | G1 | *P. persica* (peach) |
| UDP96-005 | GTAACGCTCGCTACCACAAA | CCTGCATATCACCACCCAG | G1 | *P. persica* (peach) |
| pchgms3 | ACGGTATGTCCGTACACTCTCCATG | CAACCTGTGATTGCTCCTATTAAAC | G1 | *P. persica* (peach) |
| BPPCT027 | CTCTCAAGCATCATGGGC | TGTTGCCCGGTTGTAATATC | G1 | *P. persica* (peach) |
| CPDCT005 | TTCAAGGAGAAGGCCTGAAA | ATTGTGGGTTCCAACCAATG | G1 | *P. dulcis* (almond) |
| EPDCU5100 | CTCTTCTCGCCTCCCAATTT | TGCTTAGCCCTGGGTACAAG | G1 | *P. dulcis* (almond) |
| UDP96-013 | ATTCTTCACTACACGTGCACG | CCCCAGACATACTGTGGCTT | G2 | *P. persica* (peach) |
| pchgms1 | GGGTAAATATGCCCATTGTGCAATC | GGATCATTGAACTACGTCAATCCTC | G2 | *P. persica* (peach) |
| BPPCT001 | AATTCCCAAAGGATGTGTATGAG | CAGGTGAATGAGCCAAAGC | G2 | *P. persica* (peach) |
| CPDCT025 | GACCTCATCAGCATCACCAA | TTCCCTAACGTCCCTGACAC | G3 | *P. dulcis* (almond) |
| UDP96-003 | TTGCTCAAAAGTGTCGTTGC | ACACGTAGTGCAACACTGGC | G4 | *P. persica* (peach) |
| BPPCT010 | AAAGCACAGCCCATAATGC | GTACTGTTACTGCTGGGAATGC | G4 | *P. persica* (peach) |
| BPPCT036 | AAGCAAAGTCCATAAAAACGC | GGACGAAGACGCTCCATT | G4 | *P. persica* (peach) |
| CPDCT045 | TGTGGATCAAGAAAGAGAACCA | AGGTGTGCTTGCACATGTTT | G4 | *P. dulcis* (almond) |
| UDP97-401 | TAAGAGGATCATTTTTGCCTTG | CCCTGGAGGACTGAGGGT | G5 | *P. persica* (peach) |
| BPPCT017 | TTAAGAGTTTGTGATGGGAACC | AAGCATAATTTAGCATAACCAAGC | G5 | *P. persica* (peach) |
| UDP96-001 | AGTTTGATTTTCTGATGCATCC | TGCCATAAGGACCGGTATGT | G6 | *P. persica* (peach) |
| CPSCT012 | ACGGGAGACTTTCCCAGAAG | CTTCTCGTTTCCTCCCTCCT | G6 | *P. salicina* (plum) |
| BPPCT025 | TCCTGCGTAGAAGAAGGTAGC | CGACATAAAGTCCAAATGGC | G6 | *P. persica* (peach) |
| CPPCT033 | TCAGCAAACTAGAAACAAACC | TTGCAATCTGGTTGATGTT | G7 | *P. persica* (peach) |
| UDP98-409 | GCTGATGGGTTTTATGGTTTTC | CGGACTCTTATCCTCTATCAACA | G8 | *P. persica* (peach) |
| CPSCT018 | AGGACATGTGGTCCAACCTC | GGGTTCCCCGTTACTTTCAT | G8 | *P. salicina* (plum) |
| CPDCT034 | GAGAACCTTTTGTTTGGCCTTA | CGTCGTATTTAGTGCCGTTG | G8 | *P. dulcis* (almond) |
|  |  |  |  |  |
| **Markers excluded** | |  |  |  |
| UDP98-408 | ACAGGCTTGTTGAGCATGTG | CCCTCGTGGGAAAATTTGA |  | *P. persica* (peach) |
| CPDCT035 | TCGAAGGAGGATGAAGTTGC | ATATCACGAGGGGCAAAATG |  | *P. dulcis* (almond) |

# **Table S3. Prior distributions used for approximate Bayesian computations to infer the domestication history of almonds.**

|  | Parameter | Distribution | Lower bound | | Upper bound | |
| --- | --- | --- | --- | --- | --- | --- |
| ABC analyses set 1, 2 and 3 | *N_X_** | uniform | 50 | | 1,000 | |
|  | *N_ANC_* | uniform | | 50 | | 2,000 |
|  | *T_X-ANC_* | log uniform | 10,000 | | 2,000,000 | |
|  | *T_X-Y_* | log uniform | 10,000 | | 2,000,000 | |
|  | *T_C-X_* | log uniform | 100 | | 10,000 | |
|  | *M_X-Y_* | uniform | 0.001 | | 0.02 | |

Note: Prior distributions are uniform and log uniform with their lower and upper bound. *N_X_*: effective population size of population *X*; *T_X-ANC_*: Divergence time between population *X* and an unknown ancestral population ANC; Divergence time was calculated by multiplying the generation time estimates of *Almond* species (we assumed a generation time of 10 years.); *M_X-Y_*: migration rates from populations *X* to *Y*; *T_X-Y_*: Divergence time between populations *X* and *Y*. *T_X-C_*: Divergence time between wild population *X* and cultivated population C. * *Nx* were log-transformed for fastsimcoal2 simulations (Excoffier et al., 2021).

**Table S4: Genetic diversity estimates for the four cultivated almond (*P. dulcis*) populations determined with STRUCTURE at *K*=4.** Only individuals which were assigned to a genetic cluster with a membership proportion greater than or equal to 90% were retained, i.e. 98 accessions. The observed heterozygoty (*Ho*), the unbiased expected heterozygoty (u*He*) and the fixation index (*F*) were calculated thanks to GenAlEx v.6.503, and add-in off Excel. The allelic richness (*Ar*) and the private allelic richness (*Ap*) were calculated thanks to ADZE v.1.0. Standard deviations in brackets. All significant at (*P*=0.99)

| **Genetic clusters of *P. dulcis*** | | | ***N*** | ***A_r_*** | ***A_p_*** | ***H_o_*** | ***uH_e_*** | ***F*** |
| --- | --- | --- | --- | --- | --- | --- | --- | --- |
| **Cluster #** |  | **Origin** |  |  |  |  |  |  |
| **Cluster_1** |  | Akdamar island | 15 | 2.518 (0.166) | 1.123 (0.172) | 0.527 (0.055) | 0.554 (0.052) | 0.004 (0.052) |
| **Cluster_2** |  | Caucasus/Central Asia | 28 | 3.791 (0.181) | 1.852 (0.173) | 0.743 (0.042) | 0.818 (0.040) | 0.077 (0.025) |
| **Cluster_3** |  | South Europe | 20 | 3.250 (0.164) | 1.378 (0.148) | 0.717 (0.052) | 0.728 (0.047) | -0.006 (0.023) |
| **Cluster_4** |  | EU/North-America | 35 | 3.201 (0.115) | 1.279 (0.116) | 0.661 (0.044) | 0.745 (0.027) | 0.099 (0.049) |
| **Mean** |  |  | 24.5 | 3.190 | 1.408 | 0.662 (0.025) | 0.711 (0.023) | 0.044 (0.020) |

# **Table S5: Results of the ABC-RF algorithm used to infer the demographic history of the purple Turkish *Prunus dulcis* population (set 1, round 1, Figure S1).**

We assumed three groups of scenarios:1) no gene flow (no_GF, group 1), 2) gene flow among wild and cultivated populations only (CW), and 3) gene flow among wild and cultivated populations, among wild populations (CWWW, group 3). The most likely model is the group assuming gene flow between the crop and wild population (ten of ten).

| Replicate | g1_sc1+2+3_noGF | **g2_sc1+2+3_GF_CW** | g3__sc1+2+3_GF_CWWW | Posterior probability | Prior error rate |
| --- | --- | --- | --- | --- | --- |
| 1 | 22 | **442** | 36 | 0.959433333333333 | 5.7638 |
| 2 | 19 | **458** | 23 | 0.942366666666667 | 5.7912 |
| 3 | 19 | **466** | 15 | 0.937966666666667 | 5.8262 |
| 4 | 24 | **457** | 19 | 0.9256 | 5.8463 |
| 5 | 29 | **450** | 21 | 0.943833333333333 | 5.8275 |
| 6 | 18 | **453** | 29 | 0.9194 | 5.8175 |
| 7 | 17 | **454** | 29 | 0.954366666666667 | 5.8 |
| 8 | 27 | **448** | 25 | 0.949866666666667 | 5.7912 |
| 9 | 26 | **449** | 25 | 0.938366666666667 | 5.8038 |
| 10 | 20 | **455** | 25 | 0.947066666666667 | 5.7975 |
| Average | 22 | **453** | 25 | **0.941826666666667** | **5.8065** |
| Standard deviation | 4 | **7** | 6 | **0.0123** | **0.0235** |

# **Table S6. Results of the ABC-RF algorithm used to infer the demographic history of the purple Turkish *Prunus dulcis* population (set 1, round 2, Figure S1).**

The second round compared the three scenarios of domestication of the Turkish purple *Prunus dulcis*, assuming the most likely scenario of gene flow inferred in round 1 (i.e., gene flow between only crop and wild populations (Table S5 and Figure S2)*.* The most likely model was the group assuming an origin from *Prunus orientalis* (ten of ten).

| Replicates | sc1_CWGF | sc2_CWGF | **sc3_CWGF** | Posterior probability | Prior error rate |
| --- | --- | --- | --- | --- | --- |
| 1 | 99 | 108 | **293** | 0.68 | 29.52 |
| 2 | 97 | 95 | **308** | 0.68 | 29.37 |
| 3 | 117 | 94 | **289** | 0.69 | 29.56 |
| 4 | 79 | 120 | **301** | 0.68 | 29.46 |
| 5 | 108 | 106 | **286** | 0.66 | 29.51 |
| 6 | 108 | 126 | **266** | 0.70 | 29.35 |
| 7 | 114 | 89 | **297** | 0.65 | 29.57 |
| 8 | 96 | 96 | **308** | 0.69 | 29.57 |
| 9 | 101 | 110 | **289** | 0.68 | 29.53 |
| 10 | 101 | 112 | **287** | 0.66 | 29.48 |
| Average | 102 | 106 | **292** | **0.68** | **29.49** |
| Standard deviation | 11 | 12 | **12** | 0.02 | 0.08 |

# **Table S7. Results of the ABC-RF algorithm used to infer the demographic history of the pink Turkish *Prunus dulcis* population (set 2, round 1, Figure S1).**

We assumed two groups of scenarios:1) no gene flow (no_GF, group 1) or 2) gene flow between wild and cultivated populations only (CW, group 2). The most likely model is the group assuming gene flow between the crop and wild populations (nine of ten).

| Replicates | g1_noGF | **g2_CWGF** | Posterior probability | Prior error rate |
| --- | --- | --- | --- | --- |
| 1 | 228 | **272** | 0.61 | 14.63 |
| 2 | 242 | **258** | 0.68 | 14.50 |
| 3 | 225 | **275** | 0.68 | 14.61 |
| 4 | 214 | **286** | 0.58 | 14.57 |
| 5 | 226 | **274** | 0.69 | 14.55 |
| 6 | 250 | **250** | 0.61 | 14.61 |
| 7 | 240 | **260** | 0.65 | 14.53 |
| 8 | 222 | **278** | 0.66 | 14.64 |
| 9 | 227 | **273** | 0.56 | 14.56 |
| 10 | 229 | **271** | 0.59 | 14.54 |
| Average | 230 | **270** | **0.63** | **14.57** |
| Standard deviation | 11 | **11** | **0.05** | **0.05** |

# **Table S8. Results of the ABC-RF algorithm used to infer the demographic history of the pink Turkish *Prunus dulcis* population (set 2, round 2, Figure S1).**

The second round compared the two groups of domestication scenarios of the Turkish purple *Prunus dulcis*:1) domestication from one of three wild species (*Prunus spinosissima*, *Prunus orientalis*, and *Prunus fenzliana*) or 2) domestication from the purple Turkish *Prunus dulcis*. We assumed the most likely scenario of gene flow inferred in round 1 (i.e., gene flow between only crop and wild populations (Table S7 and Figure S1)*.* The most likely model is the group assuming the origin of pink *Prunus dulcis* from purple *Prunus dulcis* (ten of ten).

| Replicates | Group 1 (sc1 + sc2 + sc3) | **Group 2 (sc4)** | Posterior probability | Prior error rate |
| --- | --- | --- | --- | --- |
| 1 | 200 | **300** | 0.65 | 17.69 |
| 2 | 197 | **303** | 0.68 | 17.60 |
| 3 | 201 | **299** | 0.64 | 17.58 |
| 4 | 177 | **323** | 0.59 | 17.68 |
| 5 | 207 | **293** | 0.68 | 17.62 |
| 6 | 181 | **319** | 0.64 | 17.67 |
| 7 | 201 | **299** | 0.67 | 17.65 |
| 8 | 170 | **330** | 0.66 | 17.72 |
| 9 | 193 | **307** | 0.71 | 17.61 |
| 10 | 201 | **299** | 0.70 | 17.79 |
| 11 | 193 | **307** | **0.66** | **17.66** |
| 12 | 12 | **12** | 0.03 | 0.06 |

# **Table S9. Results of the ABC-RF algorithm used to infer the demographic histories of blue Northwestern cultivated almonds (*Prunus dulcis*) (Set 3, Round 1, Figure S1).**

We assumed two groups of scenarios:1) no gene flow (no_GF, group 1) or 2) gene flow between wild and cultivated populations only (CW, group 2). The most likely model was the group assuming no gene flow between the crop and wild populations (eight of ten).

| Replicate | **g1 (sc1+2+3. no_GF)** | g2 (sc1+2+3. CW_GF) | Posterior probability | Prior error rate |
| --- | --- | --- | --- | --- |
| 1 | **255** | 245 | 0.71 | 2.01 |
| 2 | **247** | 253 | 0.74 | 2.00 |
| 3 | **255** | 245 | 0.78 | 1.99 |
| 4 | **264** | 236 | 0.73 | 2.04 |
| 5 | **264** | 236 | 0.73 | 1.99 |
| 6 | **278** | 222 | 0.73 | 2.03 |
| 7 | **261** | 239 | 0.72 | 2.04 |
| 8 | **254** | 246 | 0.71 | 2.01 |
| 9 | **259** | 241 | 0.73 | 2.02 |
| 10 | **243** | 257 | 0.74 | 2.02 |
| Average | **258** | 242 | **0.73** | **2.01** |
| Standard deviation | **10** | 10 | 0.02 | 0.02 |

# **Table S10. Results of the ABC-RF algorithm used to infer the demographic histories of blue northwestern cultivated almonds (*Prunus dulcis*) (Set 3, Round 2, Figure S1).**

The second round compared three scenarios of domestication of the blue Northwest cultivated almonds (*Prunus dulcis*)*:*1) domestication from *Prunus spinosissima*, 2) *Prunus fenzliana*, or 3) *Prunus orientalis*. We assumed the most likely scenario of gene flow inferred in round 1 (i.e., no gene flow between crop and wild populations (Table S9 and Figure S1)*.* The most likely model is the group assuming the origin of the blue northwestern cultivated almonds (*Prunus dulcis*) to be purple *Prunus orientalis* (ten of ten).

| Replicate | ss_sc1_noGF | ss_sc2_noGF | **ss_sc3_noGF** | Posterior probability | Prior error rate |
| --- | --- | --- | --- | --- | --- |
| 1 | 93 | 70 | **337** | 0.76 | 39.38 |
| 2 | 88 | 42 | **370** | 0.73 | 39.16 |
| 3 | 98 | 58 | **344** | 0.71 | 38.83 |
| 4 | 70 | 53 | **377** | 0.74 | 39.25 |
| 5 | 65 | 65 | **370** | 0.75 | 39.21 |
| 6 | 78 | 69 | **353** | 0.77 | 39.33 |
| 7 | 80 | 81 | **339** | 0.77 | 39.36 |
| 8 | 85 | 57 | **358** | 0.74 | 39.23 |
| 9 | 85 | 52 | **363** | 0.74 | 39.07 |
| 10 | 77 | 68 | **355** | 0.75 | 39.24 |
| Average | 82 | 62 | **357** | **0.75** | **39.21** |
| Standard deviation | 10 | 11 | **14** | 0.02 | 0.16 |

# **Table S11. Parameter estimates inferred for the most likely scenarios of the domestication history of almonds.**

| Parameter | posterior probability  mean | posterior probability median | q5% | q95% | Variance of posterior probability | Normalized mean absolute error values |
| --- | --- | --- | --- | --- | --- | --- |
| N_ANC_ | 1020 | 1065 | 126 | 1939 | 113203 | 0.43 |
| N_cropSPA_ | 468 | 478 | 65 | 923 | 42903 | 0.50 |
| N_cropTURKPurple_ | 510 | 513 | 115 | 943 | 38571 | 0.50 |
| N_cropTURKPink_ | 461 | 353 | 63 | 987 | 53931 | 0.49 |
| N_cropUS_ | 502 | 416 | 98 | 934 | 70656 | 0.92 |
| N_wildFENZA_ | 636 | 753 | 71 | 982 | 77016 | 0.49 |
| N_wildSPINO_ | 481 | 542 | 65 | 975 | 59380 | 0.53 |
| N_wildTURCO_ | 617 | 675 | 141 | 977 | 44777 | 0.45 |
| Mutation rate | 0.001 | 0.001 | 0.0002 | 0.0010 | 0.000 | 0.41 |
| m14 | 0.004 | 0.004 | 0.0011 | 0.0098 | 0.000 | 0.73 |
| m15 | 0.005 | 0.004 | 0.0014 | 0.0094 | 0.000 | 0.70 |
| m24 | 0.005 | 0.005 | 0.0013 | 0.0095 | 0.000 | 0.72 |
| m25 | 0.006 | 0.006 | 0.0010 | 0.0099 | 0.000 | 0.71 |
| m34 | 0.005 | 0.005 | 0.0011 | 0.0098 | 0.000 | 0.76 |
| m35 | 0.005 | 0.006 | 0.0011 | 0.0099 | 0.000 | 0.71 |
| m41 | 0.005 | 0.005 | 0.0011 | 0.0093 | 0.000 | 0.76 |
| m42 | 0.005 | 0.004 | 0.0011 | 0.0099 | 0.000 | 0.71 |
| m43 | 0.006 | 0.007 | 0.0012 | 0.0100 | 0.000 | 0.74 |
| m45 | 0.005 | 0.005 | 0.0012 | 0.0094 | 0.000 | 0.72 |
| m51 | 0.005 | 0.004 | 0.0010 | 0.0099 | 0.000 | 0.74 |
| m52 | 0.004 | 0.004 | 0.0011 | 0.0093 | 0.000 | 0.76 |
| m53 | 0.006 | 0.006 | 0.0012 | 0.0099 | 0.000 | 0.76 |
| m54 | 0.005 | 0.005 | 0.0013 | 0.0097 | 0.000 | 0.80 |
| T_cropUS_cropSPA_ | 743 | 410 | 130 | 3400 | 4195 | 2.65 |
| T_cropSPA_wildOR_ | 1201 | 740 | 276 | 4770 | 7890 | 0.58 |
| T_cropTURKPURPLE_ wildOR_ | 6843 | 5270 | 1363 | 24723 | 209614 | 0.52 |
| T_wildFENZA_wildSPI_ | 1048997 | 1074851 | 43960 | 1846896 | 2061564945 | 3.77 |
| T_wildSPI_ANC_ | 1991285 | 1525270 | 192625 | 3768930 | 10752946946 | 1.42 |
| T_cropPINK_cropTURKpurple_ | 4789 | 4160 | 590 | 10981 | 92299 | 0.74 |
| T_wildOR_ANC_ | 947752 | 700,740 | 31515 | 1,980,640 | 4,832,390,525 | 2.72 |

**References**

Alcala, N., & Rosenberg, N. A. (2019). G'_ST_, Jost's D, and F_ST_ are similarly constrained by allele frequencies: A mathematical, simulation, and empirical study. *Mol Ecol, 28*(7), 1624-1636. doi:<https://doi.org/10.1111/mec.15000>

Csilléry, K., François, O., & Blum, M. G. B. (2012). abc: an R package for approximate Bayesian computation (ABC). *Methods in Ecology and Evolution, 3*(3), 475-479. doi:<https://doi.org/10.1111/j.2041-210X.2011.00179.x>

Excoffier, L., Marchi, N., Marques, D. A., Matthey-Doret, R., Gouy, A., & Sousa, V. C. (2021). fastsimcoal2: demographic inference under complex evolutionary scenarios. *Bioinformatics, 37*(24), 4882-4885. doi:10.1093/bioinformatics/btab468

Pérez de los Cobos, F., Coindre, E., Dlalah, N., Quilot-Turion, B., Batlle, I., Arús, P., . . . Duval, H. (2023). Almond population genomics and non-additive GWAS reveal new insights into almond dissemination history and candidate genes for nut traits and blooming time. *Horticulture research, 10*(10). doi:10.1093/hr/uhad193
